# Manganese in Plants: From Acquisition to Subcellular Allocation

**DOI:** 10.3389/fpls.2020.00300

**Published:** 2020-03-26

**Authors:** Santiago Alejandro, Stefanie Höller, Bastian Meier, Edgar Peiter

**Affiliations:** Plant Nutrition Laboratory, Institute of Agricultural and Nutritional Sciences, Martin Luther University Halle-Wittenberg, Halle (Salle), Germany

**Keywords:** manganese transport, manganese uptake, manganese deficiency, manganese toxicity, intracellular distribution, Arabidopsis, rice, barley

## Abstract

Manganese (Mn) is an important micronutrient for plant growth and development and sustains metabolic roles within different plant cell compartments. The metal is an essential cofactor for the oxygen-evolving complex (OEC) of the photosynthetic machinery, catalyzing the water-splitting reaction in photosystem II (PSII). Despite the importance of Mn for photosynthesis and other processes, the physiological relevance of Mn uptake and compartmentation in plants has been underrated. The subcellular Mn homeostasis to maintain compartmented Mn-dependent metabolic processes like glycosylation, ROS scavenging, and photosynthesis is mediated by a multitude of transport proteins from diverse gene families. However, Mn homeostasis may be disturbed under suboptimal or excessive Mn availability. Mn deficiency is a serious, widespread plant nutritional disorder in dry, well-aerated and calcareous soils, as well as in soils containing high amounts of organic matter, where bio-availability of Mn can decrease far below the level that is required for normal plant growth. By contrast, Mn toxicity occurs on poorly drained and acidic soils in which high amounts of Mn are rendered available. Consequently, plants have evolved mechanisms to tightly regulate Mn uptake, trafficking, and storage. This review provides a comprehensive overview, with a focus on recent advances, on the multiple functions of transporters involved in Mn homeostasis, as well as their regulatory mechanisms in the plant’s response to different conditions of Mn availability.

## Introduction

Manganese (Mn) is an essential element in virtually all living organisms where it can fulfill two different functions: acting as an enzyme cofactor or as a metal with catalytic activity in biological clusters (Andresen et al., 2018). In humans, Mn functions as a cofactor for a variety of enzymes, including arginase, glutamine synthetase, pyruvate carboxylase, and Mn superoxide dismutase (MnSOD). But in comparison to other essential micronutrients, such as iron (Fe) and zinc (Zn), whose deficiencies in humans are responsible for major health problems, Mn deficiency in humans is rare. However, Mn poisoning may be encountered more frequently upon overexposure to this metal causing hepatic cirrhosis, polycythemia, dystonia, and Parkinson-like symptoms (Li and Yang, 2018). In plants, Mn is one of the 17 essential elements for growth and reproduction. It is needed in only small quantities by plants, but is ultimately as critical to growth as are the other nutrients. In photosynthetic organisms, Mn is an essential element of the metalloenzyme cluster of the oxygen-evolving complex (OEC) in photosystem II (PSII). In spite of its importance for photosynthetic activity, Mn homeostasis in plants has been poorly investigated. Nevertheless, Mn deficiency can be a serious plant nutritional disorder in soils with high pH and high partial pressure of O_2_ (pO_2_), where the bio-availability of Mn can decrease far below the level that is required for normal plant growth (Broadley et al., 2012). Fertilization with Mn salts at soil level is often not effective, since soluble Mn (Mn^2+^) is rapidly converted to plant-unavailable Mn oxides, particularly in sandy alkaline soils. (In this review, we use the general term “Mn,” unless we refer to a specific oxidation state). However, it has been argued that the application of Mn fertilizers to the soil can be an effective way to alleviate Mn deficiencies, but only if soil pH is also corrected (White and Greenwood, 2013). Foliar Mn application can supply sufficient Mn to overcome Mn deficiency, but this strategy is expensive and often impractical for farmers on marginal lands. Moreover, foliar Mn sprays are only effective for a limited time period since Mn is very little mobile in the plant and does not remobilize from older leaves to Mn-deficient young leaves (Li et al., 2017). On the other extreme, Mn toxicity can occur in poorly drained and in strongly acidic soils, where it is usually associated with other acidity-related soil fertility problems, such as aluminum toxicity and deficiencies of calcium (Ca), magnesium (Mg), and molybdenum (Mo) (Goulding, 2016).

Depending on Mn availability, plants either need to efficiently acquire and utililize Mn under limiting conditions, or to detoxify the metal under superfluous supply. Transport processes are at the core of those adaptations. In the past, Mn transporters have been identified at the molecular level in many eukaryotic organisms (Pittman, 2005). Furthermore, recent progress in Mn homeostasis has mainly focused on Arabidopsis and rice, which represent dicot and graminaceous monocot plants, respectively (Socha and Guerinot, 2014; Shao et al., 2017). Only recently, transporters involved in uptake and subcellular distribution of Mn have been characterized in a wider range of plant species. This article reviews the current knowledge of Mn uptake, translocation and subcellular distribution, as well as the functions of Mn in different plant species. Table 1 lists the Mn transporters discussed in the text.

**TABLE 1:** Manganese transport proteins reviewed in this article.

**Family/name**	**Organ(s)/tissue(s) expression**	**Subcellular localization**	**Gene expression response**	**Other substrates**	**References**
**CaCA**					
AtCAX2	Root (stele and root tip), stem, leaf, flower, pollen, fruit	Tonoplast	Unaffected by +Cu, +Mn, +Zn	Ca, Cd	Hirschi et al., 2000; Shigaki et al., 2003; Pittman et al., 2004; Edmond et al., 2009
AtCAX4	Root, stem, leaf, flower, fruit	Tonoplast	Up-regulated by +Mn, +Ni, -Ca	Cd, Ca, Zn	Cheng et al., 2002; Koren’kov et al., 2007; Mei et al., 2009
AtCAX5	Root, stem, leaf, flower, fruit	Tonoplast	Up-regulated by +Mn down-regulated by +Zn	Ca	Edmond et al., 2009
HvCAX2	Root, leaf, seed	–	Up-regulated by +Ca, +Na unaffected by +Mn	Ca	Edmond et al., 2009
LeCAX2	Root, leaf, fruit	–	–	Ca	Edmond et al., 2009
OsCAX1a	Root, shoot, flower, seed	Tonoplast	–	Ca	Kamiya et al., 2005
OsCAX3	Root, shoot, flower, seed	–	–	Ca	Kamiya et al., 2005
OsCAX4	Root	–	Up-regulated by +Ca	Ca,Cu	Yamada et al., 2014
VvCAX3	Root, stem, leaf, fruit	Tonoplast	Up-regulated by +Ca, +Na unaffected by +Mn	Ca, Cu, Li, Na	Martins et al., 2017
AtCCX3	Root, stem, leaf, flower	Tonoplast, endomembranes	Up-regulated by +K, +Na unaffected by +Mn	K, Na	Morris et al., 2008
**BICAT**					
AtBICAT1/PAM71/CCHA1	Leaf	Chloroplast (thylakoid membrane)	Unaffected by +Mn	Ca	Schneider et al., 2016; Wang et al., 2016; Eisenhut et al., 2018; Frank et al., 2019
AtBICAT2/CMT1	Root, stem, leaf, flower, fruit	Chloroplast (inner envelope)	Down-regulated by +Mn	Ca, Mg	Eisenhut et al., 2018; Frank et al., 2019; Zhang et al., 2018
**CDF/MTP**					
AtMTP8	Root (epidermis, cortex), seed	Tonoplast	Up-regulated by +Mn -Fe	Fe	Eroglu et al., 2016, 2017
AtMTP9	–	–	Unaffected by +Mn	–	Delhaize et al., 2007; Chu et al., 2017
AtMTP10	–	–	Unaffected by +Mn	–	Delhaize et al., 2007; Chu et al., 2017
AtMTP11	Root (root tip), leaf (guard cells)	Golgi/PVC	Unaffected by +Mn	–	Delhaize et al., 2007; Peiter et al., 2007
ShMTP8	–	Tonoplast	–	–	Delhaize et al., 2003, 2007
BmMTP10	Root, leaf	Endomembranes*	Up-regulated by +Mn	–	Erbasol et al., 2013
BmMTP11	Root, leaf	Endomembranes*	Unaffected by +Mn	Ni	Erbasol et al., 2013
OsMTP8.1	Root, shoot	Tonoplast	–	–	Chen et al., 2013; Takemoto et al., 2017
OsMTP8.2	Root, shoot	Tonoplast	–	–	Takemoto et al., 2017
OsMTP9	Root (endodermis, exodermis)	Plasma membrane	Unaffected by +Mn -Mn		Ueno et al., 2015
OsMTP11	Root, shoot	Golgi/TGN	Up-regulated by +Mn +Zn +Cd +Ni	Co, Ni	Zhang and Liu, 2017; Ma et al., 2018; Tsunemitsu et al., 2018
HvMTP8.1	Root, leaf	Golgi	Down-regulated by -Mn (root) down-regulated by +Mn (shoot)	–	Pedas et al., 2014
HvMTP8.2	Root, leaf	Golgi	Down-regulated by +Mn	–	Pedas et al., 2014
PtMTP11.1	–	TGN	–	–	Peiter et al., 2007
PtMTP11.2	–	TGN	–	–	Peiter et al., 2007
PbMTP8.1	–	–	–	Fe	Hou et al., 2019
PbMTP8.2	–	–	–	Fe	Hou et al., 2019
PbMTP9	–	–	–	Fe	Hou et al., 2019
PbMTP10	–	–	–	Fe	Hou et al., 2019
PbMTP11.1	–	–	–	–	Hou et al., 2019
PbMTP11.2	–	–	–	–	Hou et al., 2019
NtMTP8.1	Root, stem, leaf, flower	–	Up-regulated by +Co +Zn unaffected by +Mn	–	Liu et al., 2019
NtMTP8.4	Stem, leaf, flower	–	Up-regulated by +Cd +Co +Mn (root) up-regulated by +Cd +Co +Mg +Zn (shoot)	–	Liu et al., 2019
NtMTP11.1	Root, stem, leaf, flower	–	Up-regulated by +Co +Cd +Mn +Zn	–	Liu et al., 2019
CsMTP9	Root (endodermis)	Plasma membrane	Up-regulated by +Cd +Mn +Ni down-regulated by –Mn -Zn	Cd	Migocka et al., 2015
**NRAMP**					
AtNRAMP1	Root (Cortex, endodermis) > > shoot	Plasma membrane	Up-regulated by -Fe -Mn	Cd, Fe	Curie et al., 2000; Thomine et al., 2000; Cailliatte et al., 2010; Castaings et al., 2016
AtNRAMP2	Root (pericycle, root tip), leaf vasculature, flower, trichome	TGN	Up-regulated by -Mn down-regulated by -Fe	–	Curie et al., 2000; Alejandro et al., 2017; Gao et al., 2018
AtNRAMP3	Root (stele), leaf vasculature, developing seed	Tonoplast	Up-regulated by -Fe unaffected by -Mn	Cd, Fe	Thomine et al., 2000, 2003; Lanquar et al., 2005, 2010
AtNRAMP4	Root (stele), leaf vasculature, developing seed	Tonoplast	Up-regulated by -Fe unaffected by -Mn	Cd, Fe	Thomine et al., 2000; Lanquar et al., 2005, 2010
OsNRAMP3	Node, leaf vasculature	Plasma membrane	Unaffected by +Mn -Mn	–	Yamaji et al., 2013; Yang et al., 2013
OsNRAMP5	Root (exodermis, endodermis, stele), panicle	Plasma membrane	Up-regulated by -Fe (shoot) up-regulated by -Fe, -Zn (root) unaffected by -Mn	Cd, Fe	Ishimaru et al., 2012; Sasaki et al., 2012; Yang et al., 2014
OsNRAMP6	Leaves	Plasma membrane	–	Fe	Peris-Peris et al., 2017
HvNRAMP5	Root (epidermis, stele)	Plasma membrane	Up-regulated by -Fe unaffected by +Mn	Cd	Wu et al., 2016
BnNRAMP1b	Root, shoot	–	Up-regulated by +Cd	Cd, Zn	Meng et al., 2017
TcNRAMP3	–	Tonoplast	–	Cd, Fe	Oomen et al., 2009
TcNRAMP4	–	Tonoplast	–	Cd, Fe, Zn	Oomen et al., 2009
TcNRAMP3 (*T. cacao*)	Root, shoot	–	Up-regulated by -Fe unaffected by -Mn	Fe	Ullah et al., 2018
TcNRAMP5 (*T. cacao)*	Root	–	Up-regulated by -Fe unaffected by -Mn	Cd, Fe, Zn	Ullah et al., 2018
TcNRAMP6 (*T. cacao)*	Root, shoot	–	–	–	Ullah et al., 2018
LeNRAMP1	Root	Endomembranes*	up-regulated by -Fe	–	Bereczky et al., 2003
LeNRAMP3	Root, shoot	Endomembranes*	Up-regulated by -Fe	–	Bereczky et al., 2003
AhNRAMP1	Root, stem	–	Up-regulated by -Mn, -Zn	Zn	Wang et al., 2019
MbNRAMP1	Root	Endomembranes*	Up-regulated by -Fe	Fe	Xiao et al., 2008
**P-TYPE ATPASE**					
AtECA1	Root, stem, leaf, guard cells, trichome	ER	–	Ca, Zn	Liang et al., 1997; Wu et al., 2002; Li et al., 2008
AtECA3	Root (stele), stem, leaf vasculature, guard cells, flower, fruit	Golgi	Unaffected by -Mn	Ca, Zn	Mills et al., 2007; Li et al., 2008
LeLCA1	–	ER?	–	Ca	Johnson et al., 2009
**VIT**					
AtVIT1	Root, cotyledon, developing seed	Tonoplast	–	Fe	Kim et al., 2006
OsVIT1	Leaf > > root, stem, panicle, embryo	Tonoplast	Down-regulated by -Fe	Fe, Zn	Zhang et al., 2012; Wang et al., 2017
OsVIT2	Leaf, stem, panicle, embryo	Tonoplast	Up-regulated by +Fe down-regulated by -Fe	Fe, Zn	Zhang et al., 2012
TaVIT2	Root, shoot, seed	Tonoplast	–	Fe	Connorton et al., 2017
AtMEB1	–	ER bodies	–	Fe	Yamada et al., 2013
AtMEB2	–	ER bodies	–	Fe	Yamada et al., 2013
**YSL**					
OsYSL2	Root (phloem), leaf, vascular bundle, developing seed	Plasma membrane	Up-regulated by -Fe down-regulated by -Mn	Fe	Koike et al., 2004; Yang et al., 2014
OsYSL6	Root, shoot	Plasma membrane	Unaffected by +Mn -Mn	–	Sasaki et al., 2011
HvYSL2	Root (endodermis), shoot	–	Up-regulated by -Fe	Fe, Zn, Co, Ni, Cu	Araki et al., 2011
**ZIP**					
AtIRT1	Root (epidermis, cortex) > > shoot	Plasma membrane	Up-regulated by -Fe	Fe, Zn, Co, Ni	Korshunova et al., 1999; Curie et al., 2000; Thomine et al., 2000; Vert et al., 2002
AtZIP1	Root (stele), leaf vasculature	Tonoplast	Up-regulated by -Fe -Zn (root) up-regulated by -Mn (shoot) down-regulated by -Zn (shoot)	Zn	Milner et al., 2013
AtZIP2	Root (stele)	Plasma membrane	Down-regulated by -Fe -Mn	Zn	Milner et al., 2013
AtZIP5	Root, shoot	–	–	–	Milner et al., 2013
AtZIP6	Root, shoot	–	–	–	Milner et al., 2013
AtZIP7	Shoot > > root	–	–	Fe, Zn	Milner et al., 2013; Fu et al., 2017
AtZIP9	Root, shoot	–	–	–	Milner et al., 2013
HvIRT1	Root (epidermis, cortex**, endodermis**, pericycle), seed	Plasma membrane	Up-regulated by -Fe, -Mn	Fe, Zn	Pedas et al., 2008; Long et al., 2018
LeIRT1	Root, flower	–	–	Cd, Fe, Zn	Eckhardt et al., 2001
LeIRT2	Root	–	–	Cd, Fe, Zn	Eckhardt et al., 2001
MtZIP4	Root***, leaf	–	Down-regulated by -Fe -Mn up-regulated by +Zn	–	López-Millán et al., 2004
MtZIP7	Leaf	–	Unaffected by -Mn	–	López-Millán et al., 2004
PtIRT1	Root, leaf	–	Down-regulated by -Mn	Fe, Zn	Fu et al., 2017

+ excess, − deficiency, * in yeast cells, ** only under +Fe, *** only under +Zn.

## Functions of Manganese in Plants

Mn plays a role in diverse processes of a plant’s life cycle such as photosynthesis, respiration, scavenging of reactive oxygen species (ROS), pathogen defense, and hormone signaling. In Arabidopsis, 398 enzymes are predicted to contain Mn in the metal-binding site (The UniProt Knowledgebase^1^). Among them, 20% showed an experimental evidence to require Mn as a cofactor. In many enzymes, Mn is interchangeable with other divalent cations such as Ca, cobalt (Co), copper (Cu), Mg, or Zn. In plants, only the OEC in PSII, MnSOD, and oxalate oxidase have been shown to require exclusively Mn.

The most-well-studied function in plant metabolism that depends on Mn is the water-splitting reaction in PSII, which is the first step of photosynthesis. This process requires the tetra-Mn cluster Mn_4_O_5_Ca to split two water molecules into four electrons, four protons, and molecular O_2_ (Bricker et al., 2012). However, the delivery of Mn^2+^ and Ca^2+^ to the OEC reaction center in land plants is still under investigation. In the unicellular photosynthetic organism *Synechocystis*, PratA functions as an assembly factor and chaperone protein for efficient delivery of Mn to the PSII reaction core (Stengel et al., 2012). In green plants, it is speculated that the extrinsic protein PsbP, the closest homolog to PratA, acts as a Mn carrier protein to introduce Mn into the OEC reaction center, where it subsequently stabilizes the Mn cluster in association with PsbO and PsbQ (Bondarava et al., 2007).

Another process in plants dependent on Mn is the detoxification of ROS. In plant cells, ROS are formed mainly in chloroplasts, mitochondria, peroxisomes, and in the cytosol. Mn is a cofactor of MnSODs located in mitochondria as also in peroxisomes (Bowler et al., 1994; Corpas et al., 2017). In addition, oxalate oxidase is a Mn-dependent enzyme which catalyzes the oxidation of oxalate to CO_2_ coupled with a reduction of O_2_ to H_2_O_2_ (Requena and Bornemann, 1999). Oxalate oxidase is located in the apoplast, where it is involved in pathogen defense likely by the generation of microcidal concentrations of H_2_O_2_ and by the formation of effective barriers against pathogen penetration by H_2_O_2_-mediated lignification (Lane, 2002). Oxalate oxidase activity has been identified mainly in monocot plant species including wheat, barley, and rice (Svedruzic et al., 2005). Interestingly, germin-like proteins (GLPs) in Arabidopsis, which are homologous to oxalate oxidase, showed neither oxalate oxidase activity nor a Mn-dependent activation (Membré et al., 2000; Li et al., 2016). Intriguingly, the Mn^2+^ cation itself may act as an antioxidant molecule. In fact, it has been shown in yeast that elevated intracellular Mn was associated with a reduction of oxidative damage in yeast cells (Reddi et al., 2009). It is likely that Mn-phosphate (Mn-P) complexes act as antioxidants whereby the Mn speciation is altered by changes in phosphate concentrations (McNaughton et al., 2010).

Furthermore, Mn is an important cofactor of enzymes involved in isoprenoid biosynthesis (Wilkinson and Ohki, 1988; Köllner et al., 2008). Mn and Mg are two major cofactors of terpene synthases (Rohdich et al., 2006; Köllner et al., 2008). Therefore, it has been proposed that different patterns of plant terpene profiles are often closely correlated with Mg/Mn ratios rather than with concentrations of each cofactor element alone in the growth media (Farzadfar et al., 2017). Beside this, Mn is involved in lignin biosynthesis at two different levels: (i) Mn, besides Mg, can serve as cofactor of the phenylalanine ammonia-lyase (PAL) (Engelsma, 1972), a key enzyme in the phenylpropanoid pathway to produce monolignols; (ii) Lignin can be synthesized from monolignols that are oxidized by Mn^3+^ into monolignol radicals, which can then be added to existing phenolic groups to form lignin polymers (Önnerud et al., 2002).

Intriguingly, it has been shown that Mn can replace Mg in the active site of some enzymes of the photosynthetic pathway, including Ribulose-1,5-bisphosphate carboxylase/oxygenase (Rubisco), changing the functional role of these enzymes (Bloom and Lancaster, 2018; Bloom, 2019). Furthermore, *in vitro* experiments demonstrated that Mn is an important cofactor in abscisic acid (ABA) and auxin signaling by activating PP2C phosphatases and IAA-amino acid conjugate hydrolases, respectively (LeClere et al., 2002; Meinhard et al., 2002; Schweighofer et al., 2004). Since diverse Golgi-localized glycosyl transferases require Mn for their activity, Mn is also essential for protein glycosylation and the biosynthesis of pectin and hemicellulose polymers (Egelund et al., 2006; Strasser et al., 2007; Basu et al., 2015). Other enzymes that require Mn as a cofactor are purple acid phosphatases (PAPs) (Schenk et al., 1999; Venkidasamy et al., 2019). Also, different decarboxylases (e.g., NAD malic enzyme) and dehydrogenases (e.g., phosphoenolpyruvate carboxylase) of the tricarboxylic acid cycle can be activated by Mn, but in many cases Mn can be replaced by Mg (Burnell, 1988; Gregory et al., 2009).

Another Mn-dependent process lies in the deposition of cuticular waxes in leaves. In barley, it has been demonstrated that Mn deficiency may decrease the cuticular wax layer, which leads to a higher transpiration rate (Hebbern et al., 2009). Moreover, Mn deficiency caused a decrease of cuticular waxes in Arabidopsis leaves (Alejandro et al., 2017).

In addition, Mn plays a role in such diverse processes as chloroplast development (Rohdich et al., 2000; Hsieh et al., 2008), purine and urea catabolism (Werner et al., 2008; Cao et al., 2010), phospholipid biosynthesis (Collin et al., 1999; Nowicki et al., 2005), Ca^2+^ signaling (Kim et al., 2003; Hashimoto et al., 2012), DNA repair (Takahashi et al., 2007; Szurmak et al., 2008), or histidine biosynthesis (Glynn et al., 2005).

## Manganese Deficiency and Toxicity

In plants, Mn deficiency often occurs as a latent disorder, without clear visual symptoms. Thus, the magnitude to which Mn deficiency affects crop yield is difficult to quantify. The critical concentration for Mn deficiency is generally below 10–20 mg.kg^–1^ dry weight (Broadley et al., 2012). One of the consequences of Mn deficiency in plants is an impaired growth, leading to a decrease in biomass (Longnecker et al., 1991; Hebbern et al., 2005; Pedas et al., 2005). This can be caused by lower numbers of chloroplasts, lower net photosynthetic efficiency, and a decrease in chlorophyll content (Henriques, 2004; Hebbern et al., 2009; Alejandro et al., 2017), as well as higher susceptibility to pathogen infections (Heckman et al., 2003; Heine et al., 2011), imbalance in plant water relations (Ohki, 1985; Hebbern et al., 2009), and decreased tolerance to low temperatures (Ihnatowicz et al., 2014; Stoltz and Wallenhammar, 2014). Mn deficiency leads to a reduced number of Mn-complexes in the PSII core, which causes the destabilization and disintegration of PSII complexes that lowers the net photosynthesis rate (Schmidt et al., 2016). Besides, the disintegration of PSII complexes directly affects the thylakoid structure and promotes chlorophyll degradation leading to the development of characteristic interveinal leaf chlorosis (Papadakis et al., 2007). Due to the low phloem mobility of Mn (Ferrandon and Chamel, 1988; Li et al., 2017), typical symptoms of Mn deficiency first develop in younger leaves. Pale mottled leaves and interveinal chlorosis are the most visible symptoms of the disorder (Schmidt et al., 2016). Under severe Mn deficiency, leaves may also develop gray speck symptoms, which are characterized by brownish or necrotic spots (Broadley et al., 2012). It has been proposed that necrotic spots are a consequence of an increase in free oxygen radicals in damaged chloroplasts and a decrease in MnSOD activity (Broadley et al., 2012; Hajiboland, 2012). Similar responses have been described in Chlamydomonas, where a decrease in photosynthesis and mitochondrial MnSOD function was observed under Mn-deficient conditions (Allen et al., 2007). However, Mn-starved Arabidopsis seedlings showed a decrease in net photosynthesis while there was no loss of MnSOD activity (Lanquar et al., 2010). This suggests that the use of the cellular Mn pool for Mn-requiring metabolic reactions under low Mn conditions is variable among photosynthetic organisms.

In roots, an increase in the frequency of root hairs can be observed under Mn deficiency (Yang et al., 2008). If the deficiency becomes more severe, root tips may develop serious necrosis (Yamaji et al., 2013).

Toxic Mn concentrations are highly dependent on plant species and genotypes (Husted et al., 2009; Broadley et al., 2012; Fernando and Lynch, 2015). Excess Mn may be stored in vacuoles (Dučić and Polle, 2007; Dou et al., 2009), cell walls (Führs et al., 2010), and distributed to different leaf tissues (Fernando et al., 2006a, b). Also, Mn can be chelated in Mn-P complexes in trichomes (McNear and Küpper, 2014; Blamey et al., 2015) and complexed by organic acids in leaves (Lambers et al., 2015). Therefore, it is likely that differences between plant species lie in the cellular distribution and speciation of Mn, dominated by complexes with malate or citrate (Fernando et al., 2010). At the molecular level, excessive Mn can prevent the uptake and translocation of other essential elements such as Ca, Mg, Fe, and P (Alam et al., 2005; St. Clair and Lynch, 2005; Blamey et al., 2015; Lešková et al., 2017), inhibit chlorophyll biosynthesis (Clairmont et al., 1986; Subrahmanyam and Rathore, 2001), cause a decline in the photosynthetic rate (Nable et al., 1988; Amao and Ohashi, 2008), reduce the meristematic cell division in roots by inhibiting auxin biosynthesis (Morgan et al., 1966; Zhao et al., 2017), and lead to an increase in the accumulation of oxidized Mn and oxidized phenolic compounds in the apoplast (Fecht-Christoffers et al., 2006).

The symptoms of Mn toxicity vary widely among plant species, with chlorotic leaves and necrotic spots as the most common symptoms (Millaleo et al., 2010). Accordingly, Mn stress in plants has been explained by two main hypotheses based on either symplastic or apoplastic processes (Fernando and Lynch, 2015). The symplastic hypothesis proposes that Mn toxicity acts via photo-oxidative stress in the chloroplast that causes chlorosis. Conversely, in the apoplastic hypothesis, Mn stress damage is mainly due to the accumulation of Mn oxides, oxidized phenolic compounds, and ROS, in the cell wall, leading to necrosis. Necrotic spots have thus been associated with the accumulation and oxidation of Mn and of oxidized phenolic compounds, while chlorosis has been often attributed to Fe deficiency induced by high Mn. Surprisingly, necrotic spots are high in Mn and appear first on older leaves, whereas in chlorotic areas no Mn accumulation can be observed. It is hence conceivable that the mechanism resulting in necrotic spots differs from that which causes chlorosis.

Several lines of evidence suggest that the early target of Mn toxicity is the photosynthetic process (Millaleo et al., 2010). In fact, plants exposed to Mn excess showed a decline in net photosynthesis rate and chlorophyll content (Li et al., 2010). Although in chloroplasts the occurrence of thylakoid swelling has been associated with the administration of excess Mn (Lidon et al., 2004; Doncheva et al., 2009), the target of Mn in these photosynthetic membranes is still unclear. It has been proposed that the oxidation of Mn in chloroplasts by light-activated chlorophyll generates ROS, and thereby damages chlorophyll (Panda et al., 1987; Baldisserotto et al., 2007). At the same time, Mn may substitute Mg in chlorophyll molecules or bind to ferredoxin in the thylakoid matrix, eventually destroying the ultrastructure of chloroplasts (Panda et al., 1987; Hauck et al., 2003). Moreover, the lack of physiologically active Fe would be a secondary effect of Mn toxicity (Nian, 1989; Huang et al., 2016), which might block chlorophyll synthesis and the correct assembly of photosystem I (PSI) (Chereskin and Castelfranco, 1982; Cornah et al., 2002; Millaleo et al., 2013). Consequently, photoinhibition of PSII would likely be a late side effect of Mn exposure.

## Manganese Dynamics in Soil

Mn is one of the most abundant and widely distributed metals in nature and comprises about 0.1% of the Earth’s crust (Emsley, 2003). The element is found in minerals, combined with other elements such as oxygen, sulfur, carbon, silicon, and chlorine (Turekian and Wedepohl, 1961). Mn can exist in 11 oxidation states, ranging from –3 to +7, but in soils, Mn is mainly present as +2 (e.g., Mn^2+^), +3 (e.g., Mn_2_O_3_) and +4 (e.g., MnO_2_). Availability of Mn to plants depends on its oxidation state: Mn^2+^ is the only plant-available form and can be readily transported into root cells and translocated to the shoot, whereas the oxidized species Mn(III) and Mn(IV) form insoluble oxides that rapidly sediment (Stumm and Morgan, 1996). Thus, Mn dynamics in soils are mostly represented by the concept of a balance between soluble Mn^2+^ and insoluble Mn oxides (MnO_*x*_).

Mn deficiency in plants is particularly prevalent in alkaline soils, in which the oxidization of Mn^2+^ to unavailable MnO_*x*_ is favored. Such soils are common in the northern part of Europe, the UK, USA, China, and in southern Australia (Husted et al., 2009; George et al., 2014). In addition to pH, the oxygen level (pO_2_) in soil and soil microorganisms are also relevant factors of Mn dynamics in soils. Beside this, root exudates can modify the Mn availability in the rhizosphere. The Mn redox status in soils involves primarily the competition of soluble highly mobile oxidants, such as molecular O_2_, and soluble reducing organic molecules, derived from soil organic matter and biological sources. In aerobic soils, due to the high mobility and redox potential of O_2_, Mn oxidation is favored rather than reduction of MnO_*x*_ by microorganisms (Lovely, 1995). By contrast, waterlogging causes a reduction of MnO_*x*_ most likely by decreasing the O_2_ concentration, leading to an increase of plant-available Mn^2+^ in soil solution up to toxic levels (Khabaz-Saberi et al., 2006).

However, it has been proposed that the oxidation of Mn^2+^ in environments with abundant O_2_ is sluggish, particularly in the absence of biological catalysts, and that the oxidation of Mn^2+^ in soil is carried out predominantly by microorganisms (Sparrow and Uren, 2014). Indeed, microorganisms can catalyze Mn^2+^ oxidation in order of 10^3^ times faster than abiotic oxidation (Morgan, 2005). Some microorganisms produce enzymes which directly oxidize Mn^2+^, or produce extracellular superoxide leading to the production of insoluble MnO_*x*_ (Hansel et al., 2012; Zhang et al., 2014). Conversely, other microorganisms can reduce MnO_*x*_ and thereby increase Mn availability for plant uptake (Rengel, 2015). Also, environmental conditions (temperature, humidity) affect soil Mn availability by modulating the activity of Mn-oxidizing microorganisms (Sparrow and Uren, 2014). Therefore, the population of Mn-oxidizing and Mn-reducing microorganisms is a key factor in the availability of soil Mn for plant uptake.

Another important factor of Mn dynamics in soils is the exudation of protons (H^+^), carboxylates, and enzymes by plant roots. Proton exudation increases the Mn availability in the rhizosphere by exchanging Mn that is immobilized by negatively charged organic matter and clay minerals, and also by lowering the pH of alkaline soils (Rengel, 2015). Mn uptake by some Mn-hyperaccumlators, such as *Phytolacca* species, is based on a strong rhizosphere acidification (Lambers et al., 2015). Noteworthy, Mn availability is also increased by root exudation of carboxylates which chelate Mn and reduce Mn(IV) to Mn^2+^ in either acidic or alkaline soils (Jauregui and Reisenauer, 1982; Gherardi and Rengel, 2004).

Phytate (inositol hexaphosphate) is generally the dominant form of organic P in soils and has the potential to complex Mn^2+^ and other divalent cations. Exudation of phytases, which catalyze the degradation of phytate thus increases the Mn availability by releasing Mn^2+^ (George et al., 2014).

The release of carboxylates into the rhizosphere is a mechanism for the acquisition of not only Mn, but in particular of P. Carboxylates mobilize P absorbed to Fe/Al hydroxides by ligand exchange, especially under low P availability (Nuruzzaman et al., 2006). By contrast, elevated levels of soil-P lead to a reduction of carboxylate and phytase exudation, in turn decreasing Mn^2+^ acquisition (Lambers et al., 2015; Giles et al., 2017). However, hydroponic experiments in barley, where most likely no Mn–P complexes were formed, have also shown a decrease in Mn uptake under elevated P supply (Pedas et al., 2011). Hence, it has been proposed that there is a competition between Mn^2+^ and P during Mn uptake.

## Manganese Transport Proteins in Plants

**FIGURE 1:**
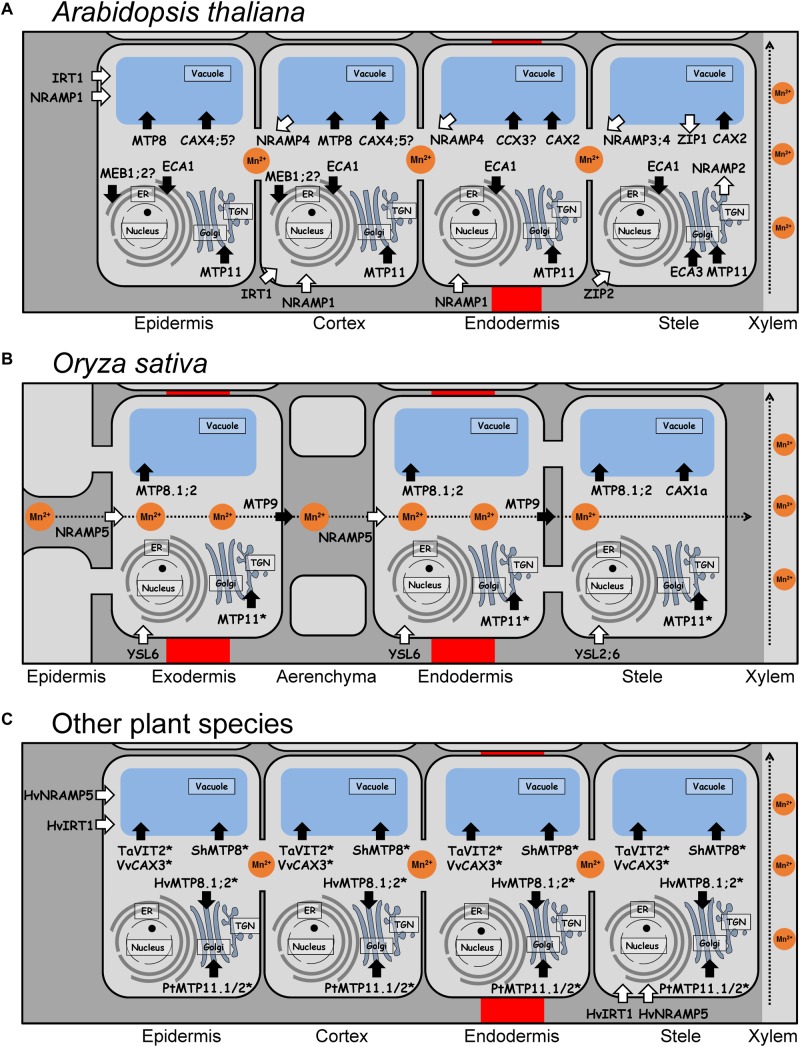
Tissue specificity and subcellular localization of Mn transport proteins in roots of different plant species. **(A)** Mn transport proteins in epidermis, endodermis, cortex, and stele (including pericycle) of Arabidopsis roots. **(B)** Mn transport proteins in exodermis, endodermis, and stele of rice roots. Radial transport of Mn^2+^ is carried out by OsNRAMP5 and OsMTP9, which are polarly localized transporters at both the exodermis and the endodermis, providing a unidirectional flux of Mn from the soil to the stele (indicated as dashed arrow). **(C)** Mn transport proteins in epidermis, endodermis, cortex, and stele of roots of other plant species. **(A–C)** White arrows indicate import into the cytosol, black arrows indicate export out of the cytosol. Transport proteins with yet unknown root tissue specificity are marked by asterisks. Proteins which subcellular localization only shown in yeast but not in plants are indicated by a question mark. Hv, *Hordeum vulgare*; Pt, *Populus trichocarpa*; Sh, *Stylosanthes hamata*; Ta, *Triticum aestivum*; Vv, *Vitis vinifera*.

As mentioned above, reduced Mn (Mn^2+^) is the only available form for plants. To maintain an optimal supply, acquisition from the rhizosphere and distribution of Mn have to be regulated. One of the most important mechanisms to regulate the acquisition from the soil is the uptake by specific transporters into root cells (Figure 1). Once Mn reaches the symplast, the main pathways for the translocation and distribution of Mn in the whole plant involve transport toward and into the xylem, transfer to the phloem, and translocation to and between the different tissues. However, most of the Mn transporters and mechanisms required for Mn loading into the xylem of the root stele, for loading Mn into the phloem, and for Mn transport into the plant cell in shoots have not been identified yet. Interestingly, the mobility of Mn in the phloem is supposed to be low (Li et al., 2017), but there is evidence that a small amount of Mn may be recycled via the phloem. In fact, it has been reported that after application of ^52^Mn to a cut barley leaf, radioactivity was detectable in the discrimination center of the shoot, in other leaves, as also in root tips (Tsukamoto et al., 2006). These results suggested that ^52^Mn was transported by unknown transporters with low affinity.

An intriguing property of Mn transport across membranes is that most of the proteins that transport Mn are not specific for the metal. Plant Mn^2+^ transporters can transport, to varying extent, other divalent cations, such as Fe^2+^, Zn^2+^, Cu^2+^, Cd^2+^, Ca^2+^, Co^2+^, and Ni^2+^. The physiological relevance of this low specificity needs further investigations, including the modification of specificity. For instance, in the Mn/Fe transporter AtMTP8, Fe transport activity was inhibited by introducing point mutations in different Fe-binding domains without affecting its Mn^2+^ transport capability (Chu et al., 2017).

**FIGURE 2:**
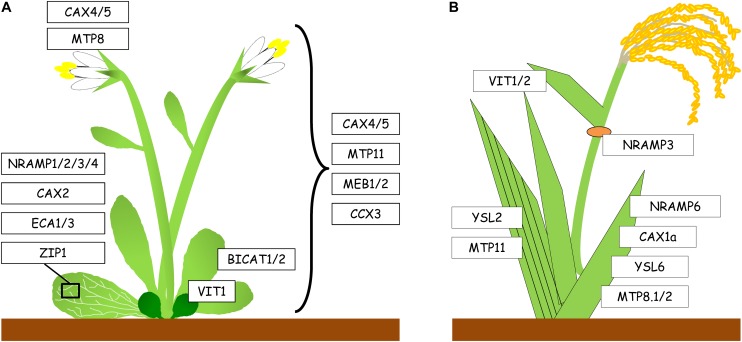
Expression of genes encoding Mn transport proteins in aerial parts of Arabidopsis and rice. **(A)** Mn transport proteins in shoots of Arabidopsis. Transporters listed on the left are expressed in the vasculature of leaves, transporters listed on the right are expressed throughout the above-ground tissues of the plant. In vegetative tissues, *AtVIT1* is only expressed in cotyledons. **(B)** Mn transport proteins in shoots of rice. *OsMTP11* and *OsYSL2* are expressed in the vasculature. *OsNRAMP3* is expressed in the first node (orange). *OsYSL6* and *OsMTP8.1/2* are expressed in older leaves, whereas expression of *OsVIT1/2* is more pronounced in younger leaves.

**FIGURE 3:**
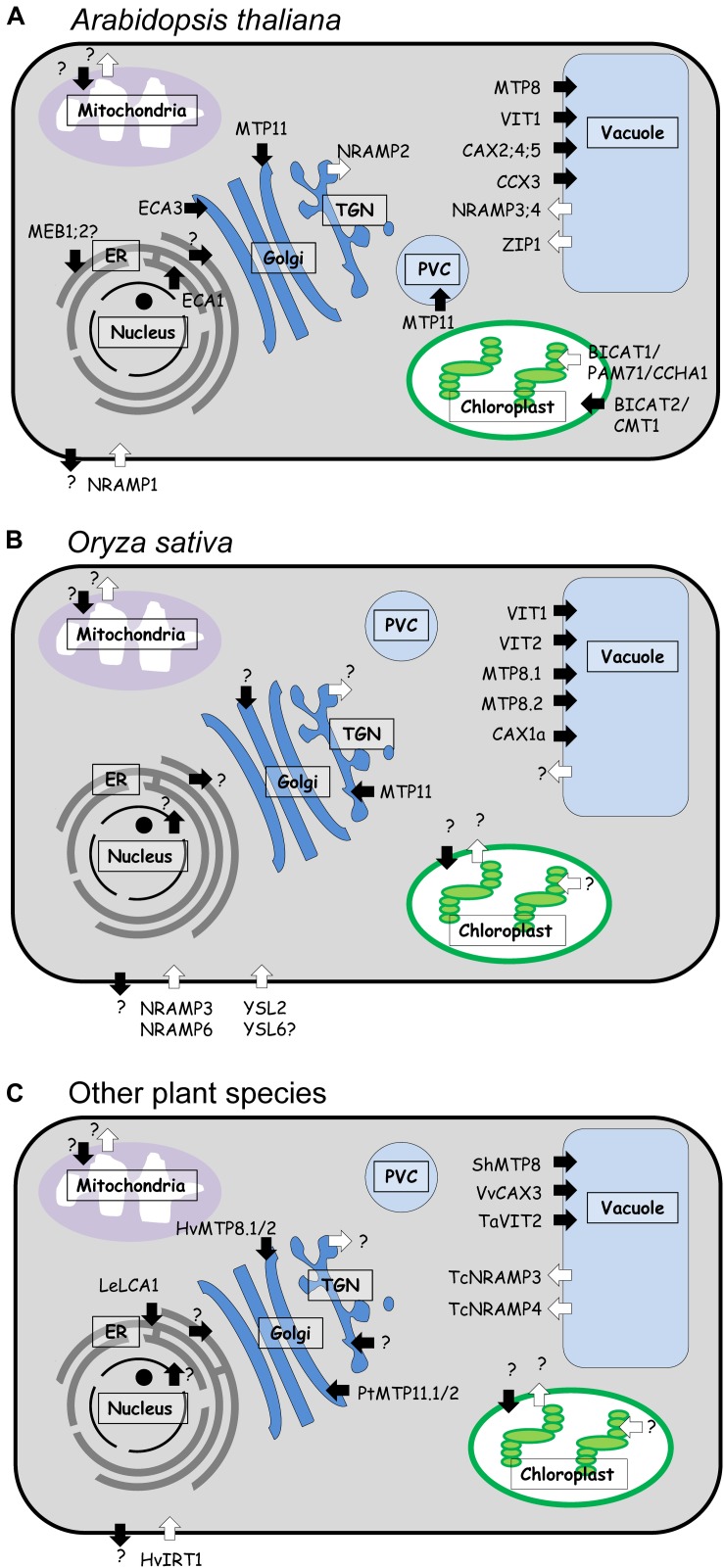
Subcellular localization of Mn transport proteins in aerial parts of different plant species. **(A)** Arabidopsis, **(B)** rice, and **(C)** other species. White arrows represent import into the cytosol, black arrows represent export from the cytosol. Transport pathways with uncharacterized Mn transporters are indicated by a question mark. Hv, *Hordeum vulgare*; Le, *Lycopersicum esculentum* (syn. *Solanum lycopersicum*); Pt, *Populus trichocarpa*; Sh, *Stylosanthes hamata*; Ta, *Triticum aestivum*; Vv, *Vitis vinifera*.

Diverse families of transport proteins are known to be components of the Mn homeostatic network in plants and can be classified into importers and exporters. Importers translocate Mn from the extracellular space or from internal compartments into the cytosol, whereas exporters are responsible for the exclusion of Mn out of the cytosol into intracellular compartments or into the apoplast. The Natural Resistance Associated Macrophage Protein (NRAMP) family, the Zinc-Regulated Transporter/Iron-Regulated Transporter (ZRT/IRT)-related Protein (ZIP) family, and the Yellow Stripe-Like (YSL) family have members involved in the transport of Mn^2+^ into the cytosol. In contrast, the Cation Diffusion Facilitator/Metal Transport Protein (CDF/MTP) family, the Vacuolar Iron Transporter (VIT) family, the Ca^2+^/Cation Antiporter (CaCA) superfamily, the Bivalent Cation Transporter (BICAT) family, and the P_2A_-type ATPase family have members involved in the transport of Mn^2+^ out of the cytosol. Figure 1 shows the tissue specificity and subcellular localization of Mn transport proteins in roots of Arabidopsis, rice, and other plant species. Genes encoding encoding Mn transport proteins in aerial parts of the plant species are depicted in Figure 2, and the subcellular localization of these proteins is displayed in Figure 3. The NRAMP family has been characterized in a number of species including bacteria, fungi, plants, and animals. These proteins act as metal/H^+^ symporters and are capable of transporting divalent metal cations (Fe^2+^, Mn^2+^, Zn^2+^, Cd^2+^, Co^2+^, Ni^2+^, Cu^2+^) into the cytosol (Nevo and Nelson, 2006), with the exceptions of OsNRAMP4 (syn. OsNrat1), that transports the trivalent cation Al^3+^ (Xia et al., 2010; Li et al., 2014), and OsNRAMP1, that appears to mediate also As(III) transport (Tiwari et al., 2014).

The ZIP transporters have been found widely in bacteria, fungi, plants, and animals and are predicted to be involved in Fe^2+^, Zn^2+^, Cd^2+^, Co^2+^, Cu^2+^, and Mn^2+^ transport. They have 8 transmembrane domains (TMD) with extracellular N- and C-termini and a cytosolic histidine-rich loop (Guerinot, 2000). YSL transporters are related to the OligoPeptide Transporter (OPT) family and are exclusively found in plants, bacteria, fungi, and archaea. Members of the YSL family are predicted to transport metals (Mn^2+^, Zn^2+^, Cu^2+^, Ni^2+^, Cd^2+^, Fe^2+^) complexed to non-proteinogenic amino acids, such as nicotianamine (NA) or phytosiderophores (PS) (Schaaf et al., 2004).

The CDF family has members in all organisms. Most CDFs are Metal^2+^/H^+^(K^+^) antiporters and mediate the efflux of Zn^2+^, Co^2+^, Fe^2+^, Cd^2+^, Ni^2+^, and/or Mn^2+^. Most of them have six TMDs with histidine-rich regions at their cytosolic N- and/or C-terminus and additionally between the 4th and 5th TMD. Based on phylogenetic relationships, the CDF family can be classified, corresponding to the main transported metal, into three major subgroups: Zn-CDFs, Fe/Zn-CDFs, and Mn-CDFs (Montanini et al., 2007).

BICAT proteins, also denominated Photosynthesis-Affected Mutant71 (PAM71) or Chloroplast Manganese Transporter (CMT) proteins, belong to the Uncharacterized Protein Family 0016 (UPF0016), which represents a new family of cation transporters present in all eukaryotes and prokaryotes, except in *Lactobacillales* and *Bacillales* (Demaegd et al., 2013, 2014; Hoecker et al., 2017). The protein structure of the plant homologs is characterized by two clusters of three transmembrane domains separated by a central loop. BICAT proteins act as Mn^2+^ and Ca^2+^ transporters (Hoecker et al., 2017; Frank et al., 2019).

CaCA transporters are present in all kingdoms of life and have a conserved core structure of ten transmembrane domains and two conserved α-repeat regions containing acidic amino acids (Pittman and Hirschi, 2016). In particular, the Cation/Ca^2+^ exchanger (CCX) and the H^+^/Cation exchanger (CAX) families, which are both members of the CaCA superfamily, are present within all plants.

VIT transporters are found in plants, fungi, and bacteria, but are absent in animals. Members of the VIT family in plants share a high degree of sequence similarity and most of them are capable to transport Fe^2+^ in addition to Mn^2+^, but their biological functions have been poorly investigated (Cao, 2019).

Plant P-type Ca^2+^-ATPases are divided into two groups, 2A and 2B, whereby the first one does not contain an N-terminal autoinhibitory domain (Johnson et al., 2009). Those P_2A_-type Ca^2+^-ATPases have also a role in Mn^2+^ transport.

## Manganese Uptake From the Soil and Translocation to the Shoot

Despite the importance of Mn in plant physiology, our knowledge of systems mediating Mn uptake followed by translocation from the roots to the shoot is limited. This is mainly due to the lack of information about the expression and subcellular localization of Mn transporters in most plants. In fact, only few Mn transporters involved in uptake and root radial transport have been identified so far. Uptake of Mn^2+^ has been assumed to be mediated by plasma membrane Ca^2+^ channels, which are generally permeable to Mn^2+^ (Wymer et al., 1997; White et al., 2002). However, this is likely to be a minor Mn^2+^ uptake pathway due to its competition with Ca^2+^, only relevant when Mn^2+^ is present in high concentrations. However, in soil, Mn^2+^ is usually far less abundant than Ca^2+^ (Broadley et al., 2012). Since this assumption, few Mn transporters have been identified mainly in *A. thaliana* and rice. Their tissue-specific localizations in roots are shown in Figure 1A for Arabidopsis, in Figure 1B for rice, and in Figure 1C for other plant species including barley, wheat, tomato, poplar, and grapevine.

In Arabidopsis, there is plenty of evidence that Mn^2+^ uptake is mainly mediated by AtNRAMP1 (Figure 1A), which is localized in the plasma membrane of epidermis and cortex cells in roots, and less in vascular tissues of young leaves (Cailliatte et al., 2010; Castaings et al., 2016). Under Mn-deficient conditions, the expression of *AtNRAMP1* is moderately up-regulated, and *nramp1* knockout mutants accumulate less Mn in shoots under Mn deficiency, which points to a function of this protein as a high-affinity Mn^2+^ uptake transporter (Cailliatte et al., 2010). *NRAMP1* orthologs in cacao, rapeseed, and peanut are also expressed in roots and complement a Mn uptake-deficient yeast strain, *smf1*, lacking a Mn transporter located in the plasma membrane (Meng et al., 2017; Ullah et al., 2018; Wang et al., 2019). In addition, active Mn^2+^ uptake may be accomplished by the ZIP transporter AtIRT1 (Castaings et al., 2016), that is considered as the major Fe uptake transporter of dicots. However, AtIRT1 is not strongly selective for Fe^2+^, but also transports Zn^2+^, Cu^2+^, Co^2+^, Ni^2+^, and Mn^2+^ (Korshunova et al., 1999). *IRT1* orthologs expressed in roots have also been identified in tomato and trifoliate orange, but their function in Mn^2+^ uptake remains to be confirmed (Eckhardt et al., 2001; Fu et al., 2017). Furthermore, two ZIP transporters expressed in the root stele are described to be involved in root-to-shoot translocation of Mn in Arabidopsis (Milner et al., 2013). AtZIP1 is a transporter localized to the tonoplast and probably involved in remobilizing Mn^2+^ from vacuoles to the cytosol in root stele cells, whereas AtZIP2 is localized to the plasma membrane and may mediate Mn^2+^ uptake into cells of the root stele. Other Mn^2+^ transporters of the AtZIP family are also expressed in roots, but their function in Mn^2+^ uptake and remobilization is still unknown (Milner et al., 2013).

In comparison with other plant species, in rice, transporters involved in uptake, xylem loading, and root-to-shoot translocation of Mn have been functionally characterized more extensively (Shao et al., 2017). The first Mn^2+^ transporter identified in rice roots was OsNRAMP5, which contributes to Mn^2+^ uptake and translocation (Sasaki et al., 2012). Further investigations showed that Mn^2+^ uptake and translocation works in conjunction with another transporter next to OsNRAMP5, namely OsMTP9 (Ueno et al., 2015). OsNRAMP5, localized to the plasma membrane of the distal side of the exo- and endodermis was shown to be responsible for the import of Mn^2+^ from the soil solution (Figure 1B). Subsequently, OsMTP9, localized to the plasma membrane of the proximal side of these cell layers, mediates the export of Mn^2+^ into the stele (Sasaki et al., 2012; Ueno et al., 2015). Knockout of either *OsMTP9* or *OsNRAMP5* significantly decreased Mn^2+^ uptake and root-to-shoot translocation, indicating that both transporters are responsible for transporting Mn^2+^ from the soil to the xylem.

Interestingly, in barley, the plasma membrane transporter HvIRT1 is implicated in the uptake and translocation of Mn^2+^, but not Fe^2+^ (Long et al., 2018). *HvIRT1* is constitutively expressed in cells of the epidermis and in pericycle funder cells (Figure 1C), but under Mn deficiency, its expression is extended to the entire pericycle and the cortex. In *hvirt1* RNAi lines, a reduced shoot Mn concentration was observed without changes in Fe or Zn concentrations. Barley is characterized as a strategy II plant species that requires HvYSL transporters for the uptake of Fe^3+^ complexed with phytosiderophores (PS) (Araki et al., 2011). Therefore, the Fe transport function of HvIRT1 has become redundant because Fe is acquired via strategy II processes. Although HvIRT1 may transport Fe in yeast (Pedas et al., 2008), HvIRT1 plays actually a key role in uptake and root-to-shoot translocation of Mn rather than Fe. HvNRAMP5 is another Mn transporter localized to the plasma membranes of cells in root tips of barley (Wu et al., 2016). In contrast to *HvIRT1*, its expression is slightly upregulated by Fe deficiency, but not by Mn deficiency. Since *HvIRT1* is up-regulated by Mn deficiency and *HvNRAMP5* is constitutively expressed in roots (Wu et al., 2016), both transporters may play different roles. Therefore, it has been suggested that HvNRAMP5 may confer constitutive Mn^2+^ uptake, while HvIRT1 plays a role in Mn^2+^ uptake only under Mn-deficient conditions (Wu et al., 2016). The barley transporter HvYSL2 appears to mediate the transport of PS complexes with metals, including Mn, in the endodermis, and it may thus be involved in the transport of minerals from the cortex to the pericycle (Araki et al., 2011). However, further analyses of HvYSL2 are required to fully understand its biological role in barley.

Once Mn has been absorbed by the root, it needs to be translocated to the shoot. To date, the molecular basis of xylem loading of Mn is still unclear, and there is no clear evidence of which Mn complex is required to translocate Mn to the shoot via the xylem. Nevertheless, transporters of the YSL family may be involved in Mn translocation. In fact, during senescence, Mn concentration decreased in Arabidopsis wild type leaves, whereas no change was observed in the *ysl1ysl3* double mutant (Waters et al., 2006). Hence, it has been proposed that both AtYSL1 and AtYSL3 are putative Mn-nicotianamine (Mn^2+^-NA) transporters, but so far, there is no direct evidence supporting this hypothesis. In rice, Mn^2+^ is transferred by OsNRAMP3 from the xylem to the phloem at the basal node, followed by its distribution to young leaves, panicles and root tips (Yamaji et al., 2013). However, at high Mn availability, Mn is distributed to mature tissues. Therefore, in rice nodes OsNRAMP3 functions as a switch for Mn distribution, whereby the protein is activated or deactivated in response to fluctuating Mn concentrations (Figure 2B). Moreover, a rice YSL transporter, OsYSL2, was proposed to be implicated in long-distance transport and distribution of Mn, since it may transport Mn^2+^-NA, as well as Fe^2+^-NA complexes (Koike et al., 2004). Because OsYSL2 is localized in phloem companion cells, it is probably involved in phloem loading of Mn^2+^-NA (Ishimaru et al., 2010), although its exact role in rice needs to be further investigated.

## Manganese Uptake by Leaf Cells

As described above, a number of plasma membrane transporters involved in Mn^2+^ import into the cytosol of root cells has been characterized. In contrast, the identity of Mn^2+^ transporters in the plasma membrane of leaf cells remains largely elusive. Recently, it has been reported that OsNRAMP6, which is expressed in shoots, is localized to the plasma membrane (Figure 3B) and functions as Fe and Mn^2+^ transporter when expressed in yeast (Peris-Peris et al., 2017). Interestingly, OsNRAMP6 rescued, to some extent, the growth of yeast strains mutated in *SMF1* (plasma membrane Mn^2+^ transporter) or *SMF2* (Mn^2+^ transporter in intracellular vesicles) under Mn-limited conditions. In plants, OsNRAMP6 accumulated in vesicles in the vicinity of the plasma membrane. Whether these vesicles represent an anterograde or retrograde trafficking stage of OsNRAMP6, depending on the Mn^2+^ status, remains to be elucidated. Moreover, an *OsNRAMP6* knock-out plant showed enhanced resistance to infection by the rice fungus *M. oryzae* and a reduced biomass compared to wild type plants (Peris-Peris et al., 2017). Therefore, it is likely that OsNRAMP6 plays a role in regulating the Mn and/or Fe contents in infected tissues which would boost the expression of defense genes. Based on the decreased root and shoot biomass of the *nramp6* mutant under non-stress conditions, it was hypothesized that OsNRAMP6 functions as a Mn uptake transporter. Future studies are needed to establish the importance of OsNRAMP6 in cellular Mn^2+^ uptake and plant growth.

A second mechanism for Mn uptake into leaf cells of rice may be conferred by the OsYSL6 transporter, which was described to transport Mn^2+^-NA complexes from the leaf apoplast to the symplast (Sasaki et al., 2011). *OsYSL6* is expressed in roots and shoots, particularly in older leaves. Due to the ability of OsYSL6 to increase Mn^2+^ influx when expressed in the *smf1* yeast mutant, it is likely localized in the plasma membrane (Sasaki et al., 2011). *ysl6* mutant plants accumulate high Mn concentrations in the apoplast of shoots and exhibit symptoms of Mn toxicity. Therefore, it is likely that OsYSL6 translocates Mn^2+^ from the apoplast to the symplast where it is sequestered under Mn excess. However, since its expression level remains unchanged under either Mn deficiency or excess, OsYSL6 may also act as a constitutive Mn importer in leaf cells.

## Intracellular Transport of Manganese

Once Mn has entered a plant cell, it must be moved to the appropriate location for the adequate supply of Mn-dependent targets or for storage. Mn is present in all cellular compartments, including ER, Golgi apparatus, mitochondria, plastids, and peroxisomes, where it performs specific cellular functions (see section Functions of Manganese in Plants), whereas vacuoles can serve as a reservoir to regulate cellular Mn homeostasis. Consequently, cells contain a plethora of transporters that are responsible for the distribution of Mn to the different compartments. Figure 3 shows an overview of Mn transporters previously described in different plant species and their subcellular localization. To structure the description of these Mn transporters in the following sections, they are organized based on their demonstrated or putative subcellular localization.

### Vacuoles as Manganese Stores

Vacuoles generally function as a primary compartment for metal internalization to avoid metal toxicity. In the case of Mn, vacuoles also serve as a temporal Mn storage pool for a proper distribution to other organelles, e.g., chloroplasts (Lanquar et al., 2010). Many transporters have been described to contribute to vacuolar Mn sequestration and unloading processes.

In the tropical legume *Stylosanthes hamata*, a plant tolerant to acidic soils in which high concentrations of plant-available Mn^2+^ can occur, the tonoplast-localized Mn transporter ShMTP8 was identified (Delhaize et al., 2003). This protein was the first characterized member of the Mn-CDF subgroup and is involved in Mn detoxification by sequestering Mn into vacuoles. Sequence analysis of *ShMTP8* showed that this protein lacks the complete N-terminal sequence and the histidine-rich loop common for members of the CDF family. When expressed in Arabidopsis, *ShMTP8* conferred tolerance to Mn toxicity (Delhaize et al., 2003). In pear and tobacco, members of the Mn-CDF subclade have also been described as Mn transporters, but their subcellular localization and relevance in Mn homeostasis is unknown (Hou et al., 2019; Liu et al., 2019). In Arabidopsis, the Mn-CDF subclade of the CDF/MTP family has four members, AtMTP8 through AtMTP11 (Montanini et al., 2007). All of them were shown to transport Mn^2+^ in yeast (Chu et al., 2017), but so far, only *AtMTP8* and *AtMTP11* have been described in more detail (Figure 3A). AtMTP8 was characterized as Mn^2+^ and Fe^2+^ transporter localized in the tonoplast (Eroglu et al., 2016; Chu et al., 2017; Eroglu et al., 2017). *AtMTP8* expression was specific to cells of the epidermis and the cortex in roots and strongly induced by Fe deficiency (Eroglu et al., 2016). Moreover, *mtp8* mutants showed chlorosis and critically low Fe levels in shoots on media with limited Fe availability, if Mn was present in the medium. This further demonstrated a Mn-specific role of AtMTP8 during Fe limitation, which lies in the detoxification of Mn taken up by the non-specific Fe transporter AtIRT1. In accord with a function of AtMTP8 in Mn detoxification, growth of *mtp8* mutants was impaired by high Mn, and *AtMTP8* expression was increased under excess Mn^2+^ supply (Eroglu et al., 2016). Besides its role in the Fe deficiency response, AtMTP8 plays a second role during seed development. An analysis of metal localization in the embryo by μXRF tomography showed that AtMTP8 is responsible for the specific accumulation of Mn in subepidermal cells on the abaxial side of the cotyledons and in cortical cells of the hypocotyl (Chu et al., 2017; Eroglu et al., 2017). In mutant embryos lacking the vacuolar Fe/Mn transporter AtVIT1, AtMTP8 built up Fe hotspots in those *AtMTP8*-expressing cell types, suggesting that AtMTP8 transports Fe in addition to Mn. This was supported by complementation of the Fe-sensitive yeast strain *ccc1*. An *mtp8vit1* double mutant showed a homogeneous distribution of both metals in all cell types of the embryo, demonstrating that both are the primary transporters determining Mn and Fe allocation (Chu et al., 2017; Eroglu et al., 2017). Mn^2+^ transport and vacuolar localization were also demonstrated for MTP8 orthologs in rice (Figure 3B). *OsMTP8.1* was highly expressed in older leaves, and it was induced and repressed by high and low Mn^2+^ levels, respectively (Chen et al., 2013). *OsMTP8.2* was expressed in roots and shoots, and it showed lower expression levels than *OsMTP8.1* (Takemoto et al., 2017). The *mtp8.1mtp8.2* double mutant suffered from necrotic leaf blades. Since Mn concentrations were comparable to those in healthy wild type plants, it has been suggested that this phenotype was the result of an insufficient Mn sequestration in the double mutant. Unlike AtMTP8 in Arabidopsis, there is no evidence that OsMTP8.1 and OsMTP8.2 are able to transport Fe or that their transcript levels are increased upon Fe deficiency (Chen et al., 2013; Takemoto et al., 2017).

VIT proteins in plants are mainly Fe transporters (Cao, 2019). AtVIT1, the most-studied VIT transporter in Arabidopsis, was identified as a vacuolar Fe transporter that is responsible for the localization of Fe primarily to the provascular strands of the embryo in seeds (Kim et al., 2006). The ability of AtVIT1 to transport Mn^2+^ was shown by metal analysis of vacuoles from the Fe-sensitive yeast strain *ccc1* expressing *AtVIT1* (Kim et al., 2006). Interestingly, seeds of a *vit1* mutant showed a localization of Fe that coincided with the localization of Mn in the subepidermal cells on the abaxial side of the cotyledons and that was dependent on AtMTP8, as discussed above (Chu et al., 2017; Eroglu et al., 2017). VIT transporters of rice, OsVIT1 and OsVIT2, not only partially rescued the Fe^2+^-sensitive phenotype, but also the Zn^2+^-sensitive phenotypes of yeast mutant strains. Similar to AtVIT1, an analysis of vacuolar metal composition of these cells showed an increased accumulation of Mn (Zhang et al., 2012). Moreover, *OsVIT1* and *OsVIT2* were shown to be highly expressed in flag leaf blades and leaf sheath. Consistent with these results, decreased accumulation of Fe and Zn was observed in flag leaves of *osvit1* and *osvit2* mutants. However, both mutants showed no significant change of Mn in these tissues or in grains (Zhang et al., 2012). These results suggest that OsVIT1 and OsVIT2 may function primarily in flag leaves to mediate vacuolar sequestration of Fe and Zn. Moreover, the Fe and Zn accumulation in seeds and the decrease of those metals in flag leaves (the source organ) of *osvit1* and *osvit2* mutants indicates an indirect involvement of both gene products in translocation of both metals to sink organs, since they are only weakly expressed in embryos, which is in contrast to *AtVIT1* (Zhang et al., 2012). Finally, there are two VIT homologs in wheat, which are located to the tonoplast (Figure 3C), but only one of them, namely TaVIT2, was shown to complement a Mn-sensitive yeast strain, *pmr1*. TaVIT2 was also able to transport Fe^2+^ and has been employed for biofortification of wheat grains with Fe (Connorton et al., 2017).

Members of the CAX family are metal transporters that mediate efflux of cations into the vacuole (Martinoia et al., 2012). All plant CAX transporters characterized to date appear to be able to transport Ca^2+^. Nevertheless, a broad metal substrate range, including Mn^2+^, is a common characteristic of these proteins. Based on amino acid sequences, the plant CAX family is divided into two clusters, type 1-A and 1-B. Their capability to transport Mn^2+^ is not related to their phylogenetic association, with *AtCAX4*, *OsCAX1a*, and *VvCAX3* being members of the type 1-A subfamily and *AtCAX2*, *AtCAX5*, *OsCAX3*, and *OsCAX4* being members of the type 1-B subfamily (Martins et al., 2017). AtCAX2 transports a range of cations into the vacuole, including Mn^2+^, Zn^2+^, and Cd^2+^ (Koren’kov et al., 2007). Knockout analysis suggested that AtCAX2 does not play a major physiological role in Ca homeostasis, but is more important for vacuolar Mn accumulation (Pittman et al., 2004). However, vacuolar Mn^2+^ transport was not completely abolished in *cax2.* AtCAX5 is a likely candidate for this residual activity. AtCAX5 showed a lower Ca^2+^ and Mn^2+^ transport activity than AtCAX2 as also a reduced Ca^2+^ transport capacity (Edmond et al., 2009). Despite the significant sequence similarity and substrate specificity of AtCAX2 and AtCAX5, a clear distinction appeared in their expression pattern and transcriptional regulation. *AtCAX5* was expressed in all tissues of Arabidopsis seedlings, particularly in the stem and the root, and at a lower level in the leaf. In addition, there was an increase in *AtCAX5* transcripts under conditions of excess Mn, and a reduction in response to excess Zn. In contrast, *AtCAX2* was detected at fairly low levels in all tissues, but was not greatly induced by any metal (Edmond et al., 2009). The relevance of these CAX transporters in intracellular Mn homeostasis is unclear. Since only the Arabidopsis *cax2* mutant showed a growth defect under Mn deficiency (Connorton et al., 2012), future genetic analysis by using *cax2cax5* double mutant plants may uncover the physiological function of these transporters in plants.

CAX2-like transporters of other species, as tomato LeCAX2 and barley HvCAX2, transport Ca^2+^ and Mn^2+^ into yeast vacuoles upon heterologous expression, but with different transport kinetics (Edmond et al., 2009). *HvCAX2* is expressed ubiquitously in roots, shoot, immature spikes, and in seeds, preferentially in the embryo rather than the endosperm. Transcripts of *HvCAX2* increased under excess Ca^2+^ and Na^+^. Likewise, *LeCAX2* was also expressed in roots and to a higher extend in leaves and fruits (Edmond et al., 2009).

*AtCAX4* was expressed at low level in all tissues, and expression increased after Mn^2+^, Na^+^, and Ni^2+^ treatment (Cheng et al., 2002). Specifically, *AtCAX4* transcript levels increased in the root apex and lateral root primordia upon Mn^2+^ and Ni^2+^ treatment and decreased if Ca^2+^ was depleted. Mutation of *AtCAX4* led to an arrested growth in seedlings under excess Cd^2+^, Mn^2+^, and auxin (Mei et al., 2009). Previously, a link between auxin-regulated plant development, cytosolic Ca^2+^, and kinases was described (Robert and Offringa, 2008). Therefore, these findings suggest that the *cax4* mutant may have increased cytosolic Ca^2+^ levels (because of reduced Ca^2+^ transport into the vacuole), that cause an impaired auxin gradient and altered root development (Mei et al., 2009). Alternatively, the auxin sensitivity phenotype may be a result of altered Mn homeostasis in *cax4* mutants, since Mn has an influence on auxin responses and auxin metabolism (Mei et al., 2009). VvCAX3 is a ubiquitously expressed vacuolar transporter for both, monovalent and divalent cations in grapevine. Expression of *VvCAX3* in yeast restored the growth on media with high Na^+^, Li^+^, Cu^2+^, and Mn^2+^ concentrations (Martins et al., 2017). Interestingly, expression of *VvCAX3* decreased during the development in parallel with Ca^2+^ accumulation in the fruit. It is likely that VvCAX3 is not only involved in Ca^2+^ sequestration, but also in the detoxification of trace elements and the mitigation of salt stress, since its transcript level increased under NaCl treatment (Martins et al., 2017).

Other members of the type 1-B subfamily of CAX transporters are *OsCAX3* and *OsCAX4* in rice. *OsCAX3* is expressed in all plant tissues, and the protein has a capacity to transport Mn^2+^. Interestingly, when expressed in yeast, next to Mn tolerance *OsCAX3* also conferred Ca^2+^ tolerance, but to a lower extent compared to other OsCAX transporters. Therefore, OsCAX3, as AtCAX2, might preferentially transport Mn^2+^, rather than Ca^2+^, into the vacuole (Kamiya et al., 2005). Nevertheless, future work is needed to confirm its vacuolar localization. By contrast, expression of *OsCAX4* provides Mn and Cu tolerance to yeast, and *OsCAX4* transcripts were increased upon prolonged salt stress *in planta* (Yamada et al., 2014). A type 1-A transporter of rice, OsCAX1a, transports Ca^2+^ and Mn^2+^ into yeast vacuoles, but is involved mainly in Ca^2+^ homeostasis in plant cells under high concentrations of Ca^2+^ (Kamiya et al., 2006). Further analysis of the function of OsCAX1a *in planta* showed that the transcript level in roots was increased by Ca^2+^ and decreased by Mn^2+^, Ni^2+^, Mg^2+^, and Cu^2+^, while shoots showed only an increase of *OsCAX1a* transcript levels after treatment with Ca^2+^. The decreased expression of *OsCAX1a* in response to other divalent cations might be a mechanism to keep high concentrations of cytosolic Ca^2+^ in order to protect the cell from toxic levels of ions like Mn^2+^ (Kamiya et al., 2006). Therefore, OsCAX1a may transport Ca^2+^ into the vacuole under Ca^2+^ toxicity or regulate Ca^2+^ homeostasis in the cytosol. In rice, further studies using combined mutants of *OsCAX1a*, *OsCAX3*, and *OsCAX4* are needed to confirm their relevance in plant Mn^2+^ and Ca^2+^ homeostasis.

Members of the CCX family were previously described as transporters of the CAX family. However, because they are phylogenetically closer to the mammalian plasma membrane K^+^-dependent Na^+^/Ca^2+^ exchangers (NCXs), this gene family, which has five members in Arabidopsis (CCX1-5), was re-classified (Pittman and Hirschi, 2016). Expression of *AtCCX3* was induced in roots and flowers upon treatment with Mn^2+^, and in yeast the expression of *AtCCX3* complemented strains defective in Mn^2+^ export (Morris et al., 2008). Tobacco cells expressing *AtCCX3* showed enhanced Mn levels, further suggesting an ability of AtCCX3 to transport Mn^2+^. The transcript level of *AtCCX3* increased in plants upon a treatment with Na^+^, K^+^, and Mn^2+^, albeit a mutation in *AtCCX3* provoked no discernible growth abnormalities under those conditions (Morris et al., 2008).

The genome of Arabidopsis contains six genes encoding NRAMP transporters, whereby AtNRAMP3 and AtNRAMP4 have been localized to the tonoplast (Thomine et al., 2003; Lanquar et al., 2005). The NRAMP homologs TcNRAMP3 and TcNRAMP4 from *Thlaspi caerulescens* were also localized to the tonoplast (Oomen et al., 2009). These NRAMP transporters from both species are involved in the transport of Cd^2+^, Fe^2+^, and Mn^2+^ (Thomine et al., 2000; Oomen et al., 2009; Pottier et al., 2015). In addition, AtNRAMP4 and TcNRAMP4 also transport Zn^2+^ when expressed in yeast (Lanquar et al., 2004; Oomen et al., 2009; Pottier et al., 2015). AtNRAMP3 and AtNRAMP4 protein levels in leaves were unaffected by Mn deficiency, but expression of *AtNRAMP4* was induced under Fe-limited conditions (Lanquar et al., 2010). Both proteins were shown to be responsible for the retrieval of Fe from vacuoles during seed germination and to mediate the export of vacuolar Mn in photosynthetic tissues of adult plants (Lanquar et al., 2005, 2010). The *nramp3nramp4* double mutant had comparable Mn concentrations in leaf mesophyll cells as wild type plants, but it showed a strong accumulation of Mn in the vacuoles (Lanquar et al., 2010). Under Mn deficiency, the double mutant showed a decreased growth which was correlated with reduced photosynthetic activity as a consequence of a shortage of Mn for the formation of OEC complexes in PSII. In contrast, *nramp3nramp4* did not show altered mitochondrial MnSOD activity under Mn deficiency (Lanquar et al., 2010). These results suggest an important role for AtNRAMP3/AtNRAMP4-dependent Mn transit through the vacuole prior to the import into chloroplasts of mesophyll cells.

### Manganese Accumulation in Chloroplasts

Mn plays an extremely important role in chloroplast function, as it is essential for photosynthetic activity. There is a high demand for Mn in the photosynthetic apparatus, mainly for the formation of the Mn_4_Ca -cluster in the OEC of PSII, which is essential for water splitting. In spite of the importance of Mn for chloroplast function, only recent studies have reported the identification and characterization of Mn transporters in chloroplast membranes. Two members of the BICAT family, which are related to the GDT1 protein in yeast (Demaegd et al., 2013) and the Mnx protein in the cyanobacterium *Synechocystis* (Brandenburg et al., 2017), are involved in Mn loading of the chloroplast and the distribution within this organelle (Figure 3A).

The Arabidopsis AtBICAT1 (syn. PAM71, CCHA1) protein is localized in the thylakoid membrane and transports Ca^2+^ and Mn^2+^ into the thylakoid lumen (Schneider et al., 2016; Wang et al., 2016; Frank et al., 2019). AtBICAT1 complemented the Mn-sensitive phenotype of the *pmr1* yeast mutant and mediated Ca^2+^ influx upon expression in *E. coli*. *bicat1* knockout mutants showed slightly pale green leaves along with compromised growth. Interestingly, growth retardation and photosynthetic activity of *bicat1* could be partially restored by exogenous treatment with Mn^2+^ (Schneider et al., 2016). In addition, transient elevations of the stromal free Ca^2+^ concentration, induced by a light-to-dark shift, were increased in *bicat1* mutants (Frank et al., 2019). Based on these findings, AtBICAT1 presumably functions in Mn^2+^ and Ca^2+^ flux into thylakoids for assembly of the Mn_4_Ca-cluster, and also in the homeostasis of stromal Ca^2+^, which regulates numerous processes in chloroplasts (Sello et al., 2018).

AtBICAT2 (syn. PAM71HL, CMT1), the second member of this family, was also described to fulfill functions in chloroplast Mn and Ca^2+^ homeostasis (Eisenhut et al., 2018; Frank et al., 2019). In contrast to AtBICAT1, it resides in the chloroplast envelope. Like *AtBICAT1*, the expression of *AtBICAT2* can also alleviate the Mn^2+^ and Ca^2+^ sensitivity phenotypes of the *pmr1* and *pmr1gdt1* yeast mutants, respectively. Disruption of *AtBICAT2* resulted in strong chlorosis, severely impaired plant growth, defective thylakoid stacking, and severe reduction of PS II complexes, resulting in diminished photosynthetic activity (Eisenhut et al., 2018; Frank et al., 2019). Consistent with a reduced oxygen evolution capacity, *bicat2* mutant chloroplasts contained less Mn than those of the wild type (Zhang et al., 2018). Also, mutants were defective in Ca^2+^ uptake across the chloroplast envelope and showed a strongly dampened dark-induced Ca^2+^ signal in the stroma (Frank et al., 2019). As consequence, the disruption of *AtBICAT2* should also lead to a decreased Ca^2+^ import into the thylakoid lumen. Taken together, these results indicate that AtBICAT2 functions as an inner envelope transporter responsible for chloroplast Mn^2+^ and Ca^2+^ uptake. Ca^2+^ dynamics and Mn concentrations in both, stroma and lumen, were likely diminished in *bicat2* mutants, which may explain the stronger phenotype compared to that of *bicat1* mutants. The phenotype of *bicat1bicat2* double mutants suggest that *AtBICAT2* is the limiting step in Mn^2+^ and Ca^2+^ delivery to the chloroplast. Further work is needed to unravel the bi-functionality and regulation of BICATs in Ca^2+^ and Mn^2+^ homeostasis in chloroplasts.

### Manganese Transport Proteins in Endomembranes

The Arabidopsis CDF protein AtMTP11 was shown to exhibit an important role in Mn detoxification. The promoter of *AtMTP11* had a high activity in root tips, shoot margins, and hydathodes, but not in epidermal cells and trichomes. Expression of *AtMTP11* in yeast complemented the Mn^2+^ hypersensitivity of the *pmr1* mutant (Peiter et al., 2007). In Arabidopsis, *mtp11* mutants were hypersensitive to elevated Mn^2+^ levels, whereas *AtMTP11-*overexpressing lines were hypertolerant (Delhaize et al., 2007; Peiter et al., 2007). As AtMTP11 appeared to be localized to the prevacuolar compartment (PVC) or to the Golgi apparatus (Figure 3A), it was suggested that it functions in the accumulation of excess Mn either into vacuoles via the PVC or in the secretion to the apoplastic space via vesicle-mediated exocytosis (Delhaize et al., 2007; Peiter et al., 2007). The latter pathway was supported by increased Mn concentrations in *mtp11* mutants (Peiter et al., 2007). Two poplar homologs of this protein, PtMTP11.1 and PtMTP11.2, were also able to complement the Mn-sensitive yeast mutant *pmr1* and targeted to the same Golgi compartments as AtMTP11 (Figure 3C; Peiter et al., 2007). Those genes are therefore likely to function in a similar way. Another MTP11 ortholog in rice, OsMTP11, was also described to be involved in Mn detoxification. *OsMTP11* is induced by high Mn and expressed specifically in conducting tissues (Zhang and Liu, 2017). Interestingly, epigenetic mechanisms (e.g., DNA methylation) were a major factor regulating the expression level of *OsMTP11* (Zhang and Liu, 2017). Knockdown of *OsMTP11* resulted in growth inhibition in the presence of high concentrations of Mn^2+^ and also led to increased accumulation of Mn in shoots and roots. By contrast, the overexpression of *OsMTP11* enhanced Mn tolerance of rice and decreased the accumulation of Mn in shoots and roots (Ma et al., 2018). Stable expression of *OsMTP11-GFP* in Arabidopsis and transient expression of this construct in rice and tobacco protoplasts showed that OsMTP11 was also located to the Golgi (Figure 3B; Farthing et al., 2017). However, recent studies suggested that OsMTP11 localized to the trans-Golgi network (TGN) when it was expressed in rice protoplasts and tobacco epidermal cells (Ma et al., 2018). Surprisingly, in tobacco epidermal cells, OsMTP11 relocalized to the plasma membrane upon treatment with high extracellular Mn^2+^ concentrations. These findings suggest that OsMTP11 is required for Mn homeostasis and contributes to Mn^2+^ tolerance in rice (Ma et al., 2018).

In the sugar beet relative *B. vulgaris* spp. *maritima*, the MTP11 homologs BmMTP10 and BmMTP11 were also characterized as Mn^2+^ transporters by complementation of the *pmr1* yeast strain (Erbasol et al., 2013). In barley, transient expression in onion epidermal cells showed that the HvMTP8.1 and HvMTP8.2 proteins, which are most closely related to the vacuolar AtMTP8 (see section Vacuoles as Manganese Stores), were localized to the Golgi apparatus and also complemented the *pmr1* strain (Pedas et al., 2014). However, the function of those CDF proteins *in planta* is still unclear.

The Arabidopsis genome encodes 15 P-type Ca^2+^-ATPases, of which the P_2A_ -type or ECA (ER-type Calcium ATPase) subfamily has two members involved in endomembrane Mn^2+^ transport (Kamrul Huda et al., 2013). AtECA1 and AtECA3 both function as a pump for Ca^2+^ and Mn^2+^, and localize to the ER and Golgi apparatus, respectively (Figure 3A). Furthermore, AtECA3 was shown to be localized also in subpopulations of endosomes or PVC (Li et al., 2008). *AtECA1* and *AtECA3* expression in the K616 yeast mutant, defective in the Golgi Ca^2+^ and Mn^2+^ pump PMR1 and in the vacuolar Ca^2+^ pump PMC1, increased its tolerance to toxic levels of Mn^2+^ and to Ca^2+^ deficiency (Liang et al., 1997; Wu et al., 2002; Mills et al., 2007). Another ER-localized ECA transporter in tomato, LCA1, also complemented the growth of K616 yeast under conditions of high Mn^2+^ and low Ca^2+^ (Johnson et al., 2009). *eca1* mutants of Arabidopsis showed impaired growth under Ca^2+^ deprivation and Mn^2+^ excess. On high-Mn^2+^ media, root hair elongation was inhibited in the mutants, suggesting an impairment in tip growth (Wu et al., 2002). Taken together, AtECA1 and LeLCA1 are likely involved in the transport of Ca^2+^ and Mn^2+^ from the cytosol into the ER. On the other hand, *eca3* was shown to be sensitive to Mn deficiency and also to Mn toxicity, but not to Ca^2+^ deficiency (Mills et al., 2007; Li et al., 2008). Thus, the phenotype of *eca3*, observed under Mn-deficient conditions, may be due to a reduction of the Mn content in the Golgi. Based on these studies, CDF and ECA proteins may be responsible for Mn^2+^ loading of Golgi-related vesicular compartments, for delivering Mn to Mn-dependent enzymes and/or for detoxification of Mn via a secretory pathway.

In Arabidopsis, AtNRAMP2 is located in the TGN (Figure 3A) and was shown to be involved in the intracellular allocation of Mn (Alejandro et al., 2017; Gao et al., 2018). *AtNRAMP2* is mainly expressed in the vasculature of roots and shoots and was barely induced upon Mn deficiency. Nevertheless, *nramp2* mutants showed a hypersensitivity to Mn deficiency and a reduction in Mn contents in vacuoles and chloroplasts with an accompanying reduction in PSII activity under those conditions (Alejandro et al., 2017). Surprisingly, the *nramp2nramp3nramp4* triple mutant did not exhibit higher sensitivity to Mn deficiency than the single *nramp2* and double *nramp3nramp4* mutants (Alejandro et al., 2017). This suggests that the three transporters act in the same pathway to deliver Mn to the chloroplasts.

NRAMP transporters from tomato (LeNRAMP1 and LeNRAMP3) and apple (MbNRAMP1) were also able to transport Mn^2+^ and located to intracellular vesicles when expressed in yeast cells, but their function in Mn homeostasis is still unclear (Bereczky et al., 2003; Xiao et al., 2008).

ER bodies are fusiform compartments connected to the ER that were found specifically in Brassicales (Matsushima et al., 2003). It has been proposed that ER bodies might play a role in the defense against pathogens and herbivores (Yamada et al., 2011). In Arabidopsis, two VIT transporters, AtMEB1 (Membrane protein of Endoplasmic reticulum Body) and AtMEB2, that localize to the ER body membrane but not to the ER network, have been identified (Yamada et al., 2013). Heterologous expression of *AtMEB1* and *AtMEB2* in yeast suppressed Fe and Mn toxicity, suggesting that they possibly act as metal transporters. ER bodies are present in hypocotyls of seedlings where they disappear during plant development. In contrast, in roots, ER bodies are constitutively present. Therefore, it has been suggested that MEB transporters may be involved in the sequestration of metals into root ER bodies under metal stress conditions (Yamada et al., 2013).

### Manganese Homeostasis in Other Organelles

In mitochondria, Mn is crucial for the activity of MnSOD which scavenges ROS generated within the citric acid cycle and the electron transport chain. However, the transport mechanisms for Mn^2+^ in plant mitochondria still remain to be elucidated. To date, no transporter involved in Mn^2+^ transport to and from plant mitochondria has been characterized. Nevertheless, in humans, a mitochondrial Ca^2+^ uniporter (MCU) that is responsible for Ca^2+^ and Mn^2+^ loading into the mitochondria has been described (Kirichok et al., 2004). Arabidopsis has six MCU isoforms which are predicted to be localized in mitochondria, except one, cMCU, that is targeted to the chloroplast (Stael et al., 2012). The cMCU protein mediates Ca^2+^ fluxes across the chloroplast envelope (Teardo et al., 2019). In humans, MCU is part of a complex named MCUC (MCU complex) which includes other subunits, including the EF hand-containing proteins MICU1 and MICU2 (Ca^2+^-sensing inhibitory subunit). MICU1 plays a decisive role in ion specificity of MCU, allowing it to distinguish between Ca^2+^ vs. Mn^2+^ (Kamer et al., 2018). In Arabidopsis, a MICU homolog, AtMICU, that binds Ca^2+^ and localizes to the mitochondria, was described (Wagner et al., 2015). These findings provoke the idea that a conserved uniporter system, similar to MCUC in humans, may mediate Ca^2+^ and Mn^2+^ uptake by plant mitochondria.

In peroxisomes, Mn is important as a cofactor of the peroxisomal MnSOD. This enzyme is present in some plant species, including pea, cucumber, and pepper (Corpas et al., 2017). However, so far, transporters to load Mn into the peroxisomes are still missing.

## Conclusion and Perspectives

The relevance of manganese as a micronutrient of plants is still largely underestimated. For a long time, the accepted dogma among animal and plant biologists has been that the physiological requirement for Mn by living cells is low and that Mn uptake exceeds the requirement. However, in natural and in agricultural settings, Mn availability can be a seriously limiting factor for plant growth, which necessitates the operation of high-affinity Mn transporters in roots and efficient mechanisms of Mn distribution in the plant to cope with Mn shortage. Crops with improved Mn uptake capacity and Mn use efficiency will achieve higher growth and yield under suboptimal Mn availability, primarily by providing sufficient Mn to PSII and thus increasing their photosynthetic efficiency.

There are many unresolved issues in our understanding of Mn transport and homeostasis, such as the transport of Mn into the xylem – Is it mediated by a vesicle-based secretory mechanism or by plasma membrane-bound exporters? How Mn is imported by different cell types in the shoot is another open question. Most Mn^2+^ transporters are not specific, but also able to move other divalent cations, like Ca^2+^, Fe^2+^, Zn^2+^, or Cu^2+^, but so far it is still unclear if and how they discriminate between the cations. In humans, the substrate selectivity of a mitochondrial Mn transporter is altered by interacting EF hand proteins (Kamer et al., 2018). In this respect, the ability of diverse Ca^2+^ transport proteins to also permeate Mn^2+^ (or vice versa) is particular interesting. The possibility that Ca^2+^ and Mn^2+^ share transport pathways mediated by ECAs, BICATs, CAXs, and Ca^2+^ channels, implies an interference of Mn^2+^ in Ca^2+^ signaling. In fact, Mn^2+^ has been demonstrated to act as a signaling agent *per se* in humans (Wang et al., 2018).

The sensing and signaling of the plant’s Mn status is another area yet to be explored: On which level is Mn transport activity regulated by the nutritional status of the plant? This review pointed out some transcriptional changes of Mn transporter-encoding genes under Mn-toxic and Mn-deficient conditions, but the signaling networks that underlie these changes are still missing. Furthermore, a recent systems-wide analysis indicated that Mn deficiency responses are primarily regulated at the posttranscriptional level (Rodríguez-Celma et al., 2016). Moreover, our understanding of how Mn transport proteins are regulated is very rudimentary. In this respect, a couple of proteins that regulate the subcellular localization of AtNRAMP1 have been identified, revealing links to inositol phosphate and choline homeostasis (Agorio et al., 2017; Gao et al., 2017). As another fascinating case, it has been demonstrated that phosphorylation, ubiquitination, and metal binding modify the stability and localization of AtIRT1, an Fe^2+^ transporter that contributes to the uptake of Mn^2+^ (Dubeaux et al., 2018). However, for the vast majority of Mn transporters, posttranslational modifications and protein-protein interactions are unknown.

Future research has to be focused on all these fundamental aspects. The function, regulation, and co-operation of Mn^2+^ transport proteins in different plant tissues upon Mn deficiency and excess ought to be understood at transcriptional and at protein level. Such studies will eventually elucidate the mechanisms controlling Mn acquisition, subcellular compartmentation, and homeostasis in plants, a knowledge that can be harnessed to develop Mn-efficient germplasm of staple crops.

## Author Contributions

SA, SH, and BM drafted the manuscript. SA, SH, BM, and EP finalized the manuscript, approved the final version of the manuscript, and agreed to be accountable for all aspects of the work.

## Conflict of Interest

The authors declare that the research was conducted in the absence of any commercial or financial relationships that could be construed as a potential conflict of interest.

## Footnotes

1 http://www.uniprot.org, accessed 20 October 2019

## References

[B1] Agorio A. Giraudat J. Bianchi M. W. Marion J. Espagne C. Castaings L. (2017). Phosphatidylinositol 3-phosphate–binding protein AtPH1 controls the localization of the metal transporter NRAMP1 in *Arabidopsis*. *Proc. Natl. Acad. Sci. U.S.A.* 114 E3354–E3363. 10.1073/pnas.1702975114 28373552 PMC5402440

[B2] Alam S. Akiha F. Kamei S. Huq S. M. I. Kawai S. (2005). Mechanism of potassium alleviation of manganese phytotoxicity in barley. *J. Plant Nutr.* 28 889–901. 10.1081/pln-200055572

[B3] Alejandro S. Cailliatte R. Alcon C. Dirick L. Domergue F. Correia D. (2017). Intracellular distribution of manganese by the trans-Golgi network transporter NRAMP2 is critical for photosynthesis and cellular redox homeostasis. *Plant Cell* 29 3068–3084. 10.1105/tpc.17.00578 29180598 PMC5757278

[B4] Allen M. D. Kropat J. Tottey S. Del Campo J. A. Merchant S. S. (2007). Manganese deficiency in *Chlamydomonas* results in loss of photosystem II and MnSOD function, sensitivity to peroxides, and secondary phosphorus and iron deficiency. *Plant Physiol.* 143 263–277. 10.1104/pp.106.088609 17085511 PMC1761973

[B5] Amao Y. Ohashi A. (2008). Effect of Mn ion on the visible light induced water oxidation activity of photosynthetic organ grana from spinach. *Catal. Commun.* 10 217–220. 10.1016/j.catcom.2008.08.022

[B6] Andresen E. Peiter E. Küpper H. (2018). Trace metal metabolism in plants. *J. Exp. Bot.* 69 909–954. 10.1093/jxb/erx465 29447378

[B7] Araki R. Murata J. Murata Y. (2011). A novel barley yellow stripe 1-like transporter (HvYSL2) localized to the root endodermis transports metal-phytosiderophore complexes. *Plant Cell Physiol.* 52 1931–1940. 10.1093/pcp/pcr126 21937676

[B8] Baldisserotto C. Ferroni L. Anfuso E. Pagnoni A. Fasulo M. P. Pancaldi S. (2007). Responses of *Trapa natans* L. floating laminae to high concentrations of manganese. *Protoplasma* 231 65–82. 10.1007/s00709-007-0242-2 17602280

[B9] Basu D. Tian L. Wang W. Bobbs S. Herock H. Travers A. (2015). A small multigene hydroxyproline-*O*-galactosyltransferase family functions in arabinogalactan-protein glycosylation, growth and development in Arabidopsis. *BMC Plant Biol.* 15:295. 10.1186/s12870-015-0670-7 26690932 PMC4687291

[B10] Bereczky Z. Wang H. Y. Schubert V. Ganal M. Bauer P. (2003). Differential regulation of *nramp* and *irt* metal transporter genes in wild type and iron uptake mutants of tomato. *J. Biol. Chem.* 278 24697–24704. 10.1074/jbc.m301365200 12709425

[B11] Blamey F. P. C. Hernandez-Soriano M. Cheng M. Tang C. Paterson D. Lombi E. (2015). Synchrotron-based techniques shed light on mechanisms of plant sensitivity and tolerance to high manganese in the root environment. *Plant Physiol.* 169 2006–2020. 10.1104/pp.15.00726 26395840 PMC4634059

[B12] Bloom A. J. (2019). Metal regulation of metabolism. *Curr. Opin. Chem. Biol.* 49 33–38. 10.1016/j.cbpa.2018.09.017 30296690

[B13] Bloom A. J. Lancaster K. M. (2018). Manganese binding to Rubisco could drive a photorespiratory pathway that increases the energy efficiency of photosynthesis. *Nat. Plants* 4 414–422. 10.1038/s41477-018-0191-0 29967515

[B14] Bondarava N. Un S. Krieger-Liszkay A. (2007). Manganese binding to the 23 kDa extrinsic protein of photosystem II. *Biochim. Biophys. Acta Bioenerg.* 1767 583–588. 10.1016/j.bbabio.2007.01.001 17292849

[B15] Bowler C. Van Camp W. Van Montagu M. Inzé D. Asada K. (1994). Superoxide dismutase in plants. *Crit. Rev. Plant Sci.* 13 199–218.

[B16] Brandenburg F. Schoffman H. Kurz S. Krämer U. Keren N. Weber A. P. M. (2017). The *Synechocystis* manganese exporter Mnx is essential for manganese homeostasis in cyanobacteria. *Plant Physiol.* 173 1798–1810. 10.1104/pp.16.01895 28153926 PMC5338678

[B17] Bricker T. M. Roose J. L. Fagerlund R. D. Frankel L. K. Eaton-Rye J. J. (2012). The extrinsic proteins of Photosystem II. *Biochim. Biophys. Acta Bioenerg.* 1817 121–142. 10.1016/j.bbabio.2011.07.006 21801710

[B18] Broadley M. Brown P. Cakmak I. Rengel Z. Zhao F. (2012). “Function of nutrients: micronutrients,” in *Marschner’s Mineral Nutrition of Higher Plants*, 3rd Edn, ed. Marschner P. (Oxford: Elsevier), 191–249.

[B19] Burnell J. N. (1988). “The biochemistry of manganese in plants,” in *Manganese in Soils and Plants*, eds Graham R. D. Hannam R. J. Uren N. C. (Dordrecht: Springer), 125–137.

[B20] Cailliatte R. Schikora A. Briat J.-F. Mari S. Curie C. (2010). High-affinity manganese uptake by the metal transporter NRAMP1 is essential for Arabidopsis growth in low manganese conditions. *Plant Cell* 22 904–917. 10.1105/tpc.109.073023 20228245 PMC2861449

[B21] Cao F. Q. Werner A. K. Dahncke K. Romeis T. Liu L. H. Witte C. P. (2010). Identification and characterization of proteins involved in rice urea and arginine catabolism. *Plant Physiol.* 154 98–108. 10.1104/pp.110.160929 20631318 PMC2938139

[B22] Cao J. (2019). Molecular evolution of the vacuolar iron transporter (VIT) family genes in 14 plant species. *Genes* 10:144. 10.3390/genes10020144 30769903 PMC6409731

[B23] Castaings L. Caquot A. Loubet S. Curie C. (2016). The high-affinity metal transporters NRAMP1 and IRT1 team up to take up iron under sufficient metal provision. *Sci. Rep.* 6 37222–37222. 10.1038/srep37222 27849020 PMC5110964

[B24] Chen Z. Fujii Y. Yamaji N. Masuda S. Takemoto Y. Kamiya T. (2013). Mn tolerance in rice is mediated by MTP8.1, a member of the cation diffusion facilitator family. *J. Exp. Bot.* 64 4375–4387. 10.1093/jxb/ert243 23963678 PMC3808320

[B25] Cheng N.-H. Pittman J. K. Shigaki T. Hirschi K. D. (2002). Characterization of CAX4, an Arabidopsis H^+^ /cation antiporter. *Plant Physiol.* 128 1245–1254. 10.1104/pp.010857 11950973 PMC154252

[B26] Chereskin B. M. Castelfranco P. A. (1982). Effects of iron and oxygen on chlorophyll biosynthesis. *Plant Physiol.* 69 112–116. 10.1104/pp.69.1.112 16662140 PMC426156

[B27] Chu H.-H. Car S. Socha A. L. Hindt M. N. Punshon T. Guerinot M. L. (2017). The *Arabidopsis* MTP8 transporter determines the localization of manganese and iron in seeds. *Sci. Rep.* 7 11024–11024. 10.1038/s41598-017-11250-9 28887568 PMC5591227

[B28] Clairmont K. B. Hagar W. G. Davis E. A. (1986). Manganese toxicity to chlorophyll synthesis in tobacco callus. *Plant Physiol.* 80 291–293. 10.1104/pp.80.1.291 16664602 PMC1075100

[B29] Collin S. Justin A. M. Cantrel C. Arondel V. Kader J. C. (1999). Identification of AtPIS, a phosphatidylinositol synthase from *Arabidopsis*. *Eur. J. Biochem.* 262 652–658. 10.1046/j.1432-1327.1999.00378.x 10411624

[B30] Connorton J. M. Jones E. R. Rodríguez-Ramiro I. Fairweather-Tait S. Uauy C. Balk J. (2017). Wheat vacuolar iron transporter TaVIT2 transports Fe and Mn and is effective for biofortification. *Plant Physiol.* 174 2434–2444. 10.1104/pp.17.00672 28684433 PMC5543970

[B31] Connorton J. M. Webster R. E. Cheng N. Pittman J. K. (2012). Knockout of multiple *Arabidopsis* cation/H^+^ exchangers suggests isoform-specific roles in metal stress response, germination and seed mineral nutrition. *PLoS One* 7:e47455. 10.1371/journal.pone.0047455 23071810 PMC3470555

[B32] Cornah J. E. Roper J. M. Singh D. P. Smith A. G. (2002). Measurement of ferrochelatase activity using a novel assay suggests that plastids are the major site of haem biosynthesis in both photosynthetic and non-photosynthetic cells of pea (*Pisum sativum* L.). *Biochem. J.* 362 423–432. 10.1042/bj3620423 11853551 PMC1222403

[B33] Corpas F. J. Barroso J. B. Palma J. M. Rodriguez-Ruiz M. (2017). Plant peroxisomes: a nitro-oxidative cocktail. *Redox Biol.* 11 535–542. 10.1016/j.redox.2016.12.033 28092771 PMC5238456

[B34] Curie C. Alonso J. M. Le Jean M. Ecker J. R. Briat J.-F. (2000). Involvement of NRAMP1 from *Arabidopsis thaliana* in iron transport. *Biochem. J.* 347:749. 10.1042/0264-6021:3470749 10769179 PMC1221012

[B35] Delhaize E. Gruber B. D. Pittman J. K. White R. G. Leung H. Miao Y. (2007). A role for the *AtMTP11* gene of Arabidopsis in manganese transport and tolerance. *Plant J.* 51 198–210. 10.1111/j.1365-313x.2007.03138.x 17559518

[B36] Delhaize E. Kataoka T. Hebb D. M. White R. G. Ryan P. R. (2003). Genes encoding proteins of the cation diffusion facilitator family that confer manganese tolerance. *Plant Cell* 15 1131–1142. 10.1105/tpc.009134 12724539 PMC153721

[B37] Demaegd D. Colinet A. S. Deschamps A. Morsomme P. (2014). Molecular evolution of a novel family of putative calcium transporters. *PLoS One* 9:e100851. 10.1371/journal.pone.0100851 24955841 PMC4067407

[B38] Demaegd D. Foulquier F. Colinet A. S. Gremillon L. Legrand D. Mariot P. (2013). Newly characterized Golgi-localized family of proteins is involved in calcium and pH homeostasis in yeast and human cells. *Proc. Natl. Acad. Sci. U.S.A.* 110 6859–6864. 10.1073/pnas.1219871110 23569283 PMC3637739

[B39] Doncheva S. Poschenrieder C. Stoyanova Z. Georgieva K. Velichkova M. Barceló J. (2009). Silicon amelioration of manganese toxicity in Mn-sensitive and Mn-tolerant maize varieties. *Environ. Exp. Bot.* 65 189–197. 10.1016/j.envexpbot.2008.11.006

[B40] Dou C. M. Fu X. P. Chen X. C. Shi J. Y. Chen Y. X. (2009). Accumulation and detoxification of manganese in hyperaccumulator *Phytolacca americana*. *Plant Biol.* 11 664–670. 10.1111/j.1438-8677.2008.00163.x 19689773

[B41] Dubeaux G. Neveu J. Zelazny E. Vert G. (2018). Metal sensing by the IRT1 transporter-receptor orchestrates its own degradation and plant metal nutrition. *Mol. Cell* 69 953–964. 10.1016/j.molcel.2018.02.009 29547723

[B42] Dučić T. Polle A. (2007). Manganese toxicity in two varieties of Douglas fir (*Pseudotsuga menziesii* var. *viridis* and *glauca*) seedlings as affected by phosphorus supply. *Funct. Plant Biol.* 34 31–40. 10.1071/FP06157 32689329

[B43] Eckhardt U. Mas Marques A. Buckhout T. J. (2001). Two iron-regulated cation transporters from tomato complement metal uptake-deficient yeast mutants. *Plant Mol. Biol.* 45 437–448. 11352462 10.1023/a:1010620012803

[B44] Edmond C. Shigaki T. Ewert S. Nelson M. D. Connorton J. M. Chalova V. (2009). Comparative analysis of CAX2-like cation transporters indicates functional and regulatory diversity. *Biochem. J.* 418 145–154. 10.1042/BJ20081814 18950291

[B45] Egelund J. Petersen B. L. Motawia M. S. Damager I. Faik A. Olsen C. E. (2006). *Arabidopsis thaliana* RGXT1 and RGXT2 encode Golgi-localized (1,3)-alpha-D-xylosyltransferases involved in the synthesis of pectic rhamnogalacturonan-II. *Plant Cell* 18 2593–2607. 10.1105/tpc.105.036566 17056709 PMC1626629

[B46] Eisenhut M. Hoecker N. Schmidt S. B. Basgaran R. M. Flachbart S. Jahns P. (2018). The plastid envelope CHLOROPLAST MANGANESE TRANSPORTER1 is essential for manganese homeostasis in *Arabidopsis*. *Mol. Plant* 11 955–969. 10.1016/j.molp.2018.04.008 29734002

[B47] Emsley J. (2003). *Nature’s Building Blocks: An A-Z Guide to the Elements.* Oxford: Oxford University Press.

[B48] Engelsma G. (1972). A possible role of divalent manganese ions in the photoinduction of phenylalanine ammonia-lyase. *Plant Physiol.* 50 599–602. 10.1104/pp.50.5.599 16658225 PMC366198

[B49] Erbasol I. Bozdag G. O. Koc A. Pedas P. Karakaya H. C. (2013). Characterization of two genes encoding metal tolerance proteins from *Beta vulgaris* subspecies *maritima* that confers manganese tolerance in yeast. *Biometals* 26 795–804. 10.1007/s10534-013-9658-7 23864431

[B50] Eroglu S. Giehl R. F. H. Meier B. Takahashi M. Terada Y. Ignatyev K. (2017). Metal tolerance protein 8 mediates manganese homeostasis and iron reallocation during seed development and germination. *Plant Physiol.* 174 1633–1647. 10.1104/pp.16.01646 28461400 PMC5490884

[B51] Eroglu S. Meier B. von Wirén N. Peiter E. (2016). The vacuolar manganese transporter MTP8 determines tolerance to iron deficiency-induced chlorosis in Arabidopsis. *Plant Physiol.* 170 1030–1045. 10.1104/pp.15.01194 26668333 PMC4734556

[B52] Farthing E. C. Menguer P. K. Fett J. P. Williams L. E. (2017). OsMTP11 is localised at the Golgi and contributes to Mn tolerance. *Sci. Rep.* 7:15258. 10.1038/s41598-017-15324-6 29127328 PMC5681648

[B53] Farzadfar S. Zarinkamar F. Hojati M. (2017). Magnesium and manganese affect photosynthesis, essential oil composition and phenolic compounds of *Tanacetum parthenium*. *Plant Physiol. Biochem.* 112 207–217. 10.1016/j.plaphy.2017.01.002 28092849

[B54] Fecht-Christoffers M. M. Führs H. Braun H. P. Horst W. J. (2006). The role of hydrogen peroxide-producing and hydrogen peroxide-consuming peroxidases in the leaf apoplast of cowpea in manganese tolerance. *Plant Physiol.* 140 1451–1463. 10.1104/pp.105.070474 16489137 PMC1435823

[B55] Fernando D. R. Bakkaus E. J. Perrier N. Baker A. J. Woodrow I. E. Batianoff G. N. (2006a). Manganese accumulation in the leaf mesophyll of four tree species: a PIXE/EDAX localization study. *New Phytol.* 171 751–758. 10.1111/j.1469-8137.2006.01783.x 16918546

[B56] Fernando D. R. Batianoff G. N. Baker A. J. Woodrow I. E. (2006b). In vivo localization of manganese in the hyperaccumulator *Gossia bidwillii* (Benth.) N. Snow & Guymer (Myrtaceae) by cryo-SEM/EDAX. *Plant Cell Environ.* 29 1012–1020. 10.1111/j.1365-3040.2006.01498.x 17087483

[B57] Fernando D. R. Lynch J. P. (2015). Manganese phytotoxicity: new light on an old problem. *Annals Bot.* 116 313–319. 10.1093/aob/mcv111 26311708 PMC4549964

[B58] Fernando D. R. Mizuno T. Woodrow I. E. (2010). Characterization of foliar manganese (Mn) in Mn (hyper) accumulators using X−ray absorption spectroscopy. *New Phytol.* 188 1014–1027. 10.1111/j.1469-8137.2010.03431.x 20819177

[B59] Ferrandon M. Chamel A. R. (1988). Cuticular retention, foliar absorption and translocation of Fe, Mn and Zn supplied in organic and inorganic form. *J. Plant Nutr.* 11 247–263. 10.1080/01904168809363800

[B60] Frank J. Happeck R. Meier B. Hoang M. T. T. Stribny J. Hause G. (2019). Chloroplast-localized BICAT proteins shape stromal calcium signals and are required for efficient photosynthesis. *New Phytol.* 221 866–880. 10.1111/nph.15407 30169890

[B61] Fu X.-Z. Zhou X. Xing F. Ling L.-L. Chun C.-P. Cao L. (2017). Genome-wide identification, cloning and functional analysis of the Zinc/Iron-Regulated Transporter-Like Protein (ZIP) gene family in trifoliate orange (*Poncirus trifoliata* L. *Raf.)*. *Front. Plant Sci.* 8:588. 10.3389/fpls.2017.00588 28469631 PMC5395618

[B62] Führs H. Behrens C. Gallien S. Heintz D. Van Dorsselaer A. Braun H. P. (2010). Physiological and proteomic characterization of manganese sensitivity and tolerance in rice (*Oryza sativa*) in comparison with barley (*Hordeum vulgare*). *Ann. Bot.* 105 1129–1140. 10.1093/aob/mcq046 20237113 PMC2887067

[B63] Gao H. Xie W. Yang C. Xu J. Li J. Wang H. (2018). NRAMP2, a trans-Golgi network-localized manganese transporter, is required for *Arabidopsis* root growth under manganese deficiency. *New Phytol.* 217 179–193. 10.1111/nph.14783 28913895

[B64] Gao Y. Q. Chen J. G. Chen Z. R. An D. Lv Q. Y. Han M. L. (2017). A new vesicle trafficking regulator CTL1 plays a crucial role in ion homeostasis. *PLoS Biol.* 15:e2002978. 10.1371/journal.pbio.2002978 29284002 PMC5746208

[B65] George T. S. French A. S. Brown L. K. Karley A. J. White P. J. Ramsay L. (2014). Genotypic variation in the ability of landraces and commercial cereal varieties to avoid manganese deficiency in soils with limited manganese availability: is there a role for root-exuded phytases? *Physiol. Plant.* 151 243–256. 10.1111/ppl.12151 24438182

[B66] Gherardi M. J. Rengel Z. (2004). The effect of manganese supply on exudation of carboxylates by roots of lucerne (Medicago sativa). *Plant Soil* 260 271–282. 10.1023/b:plso.0000030182.11473.3b

[B67] Giles C. D. Brown L. K. Adu M. O. Mezeli M. M. Sandral G. A. Simpson R. J. (2017). Response-based selection of barley cultivars and legume species for complementarity: root morphology and exudation in relation to nutrient source. *Plant Sci.* 255 12–28. 10.1016/j.plantsci.2016.11.002 28131338

[B68] Glynn S. E. Baker P. J. Sedelnikova S. E. Davies C. L. Eadsforth T. C. Levy C. W. (2005). Structure and mechanism of imidazoleglycerol-phosphate dehydratase. *Structure* 13 1809–1817. 10.1016/j.str.2005.08.012 16338409

[B69] Goulding K. W. T. (2016). Soil acidification and the importance of liming agricultural soils with particular reference to the United Kingdom. *Soil Use Manag.* 32 390–399. 10.1111/sum.12270 27708478 PMC5032897

[B70] Gregory A. L. Hurley B. A. Tran H. T. Valentine A. J. She Y. M. Knowles V. L. (2009). In vivo regulatory phosphorylation of the phosphoenolpyruvate carboxylase AtPPC1 in phosphate-starved *Arabidopsis thaliana*. *Biochem. J.* 420 57–65. 10.1042/BJ20082397 19228119 PMC2677216

[B71] Guerinot M. L. (2000). The ZIP family of metal transporters. *Biochim. Biophys. Acta Biomembr.* 1465 190–198. 10.1016/s0005-2736(00)00138-3 10748254

[B72] Hajiboland R. (2012). “Effect of micronutrient deficiencies on plants stress responses,” in *Abiotic Stress Responses in Plants*, eds Ahmad P. Prasad M. (New York, NY: Springer), 283–329. 10.1007/978-1-4614-0634-1_16

[B73] Hansel C. M. Zeiner C. A. Santelli C. M. Webb S. M. (2012). Mn(II) oxidation by an ascomycete fungus is linked to superoxide production during asexual reproduction. *Proc. Natl. Acad. Sci. U.S.A.* 109 12621–12625. 10.1073/pnas.1203885109 22802654 PMC3412038

[B74] Hashimoto K. Eckert C. Anschütz U. Scholz M. Held K. Waadt R. (2012). Phosphorylation of calcineurin B-like (CBL) calcium sensor proteins by their CBL-interacting protein kinases (CIPKs) is required for full activity of CBL-CIPK complexes toward their target proteins. *J. Biol. Chem.* 287 7956–7968. 10.1074/jbc.M111.279331 22253446 PMC3318702

[B75] Hauck M. Paul A. Gross S. Raubuch M. (2003). Manganese toxicity in epiphytic lichens: chlorophyll degradation and interaction with iron and phosphorus. *Environ. Exp. Bot.* 49 181–191. 10.1016/s0098-8472(02)00069-2

[B76] Hebbern C. A. Laursen K. H. Ladegaard A. H. Schmidt S. B. Pedas P. Bruhn D. (2009). Latent manganese deficiency increases transpiration in barley (*Hordeum vulgare*). *Physiol. Plant.* 135 307–316. 10.1111/j.1399-3054.2008.01188.x 19140891

[B77] Hebbern C. A. Pedas P. Schjoerring J. K. Knudsen L. Husted S. (2005). Genotypic differences in manganese efficiency: field experiments with winter barley (*Hordeum vulgare* L.). *Plant Soil* 272 233–244. 10.1007/s11104-004-5048-9

[B78] Heckman J. R. Clarke B. B. Murphy J. A. (2003). Optimizing manganese fertilization for the suppression of take-all patch disease on creeping bentgrass. *Crop Sci.* 43 1395–1398. 10.2135/cropsci2003.1395

[B79] Heine G. Max J. F. J. Führs H. Moran-Puente D. W. Heintz D. Horst W. J. (2011). Effect of manganese on the resistance of tomato to *Pseudocercospora fuligena*. *J. Plant Nutr. Soil Sci.* 174 827–836. 10.1002/jpln.201000440

[B80] Henriques F. S. (2004). Reduction in chloroplast number accounts for the decrease in the photosynthetic capacity of Mn-deficient pecan leaves. *Plant Sci.* 166 1051–1055. 10.1016/j.plantsci.2003.12.022

[B81] Hirschi K. D. Korenkov V. D. Wilganowski N. L. Wagner G. J. (2000). Expression of *Arabidopsis CAX2* in tobacco. Altered metal accumulation and increased manganese tolerance. *Plant Physiol*. 124 125–134. 10.1104/pp.124.1.125 10982428 PMC59128

[B82] Hoecker N. Leister D. Schneider A. (2017). Plants contain small families of UPF0016 proteins including the PHOTOSYNTHESIS AFFECTED MUTANT71 transporter. *Plant Signal. Behav* 12:e1278101. 10.1080/15592324.2016.1278101 28075225 PMC5351731

[B83] Hou L. Gu D. Li Y. Li J. Li J. Chen X. (2019). Phylogenetic and expression analysis of Mn-CDF transporters in pear (*Pyrus bretschneideri* Rehd.). *Plant Mol. Biol. Report.* 37 98–110. 10.1007/s11105-019-01142-9

[B84] Hsieh M. H. Chang C. Y. Hsu S. J. Chen J. J. (2008). Chloroplast localization of methylerythritol 4-phosphate pathway enzymes and regulation of mitochondrial genes in *ispD* and *ispE* albino mutants in Arabidopsis. *Plant Mol. Biol.* 66 663–673. 10.1007/s11103-008-9297-5 18236010

[B85] Huang Y. L. Yang S. Long G. X. Zhao Z. K. Li X. F. Gu M. H. (2016). Manganese toxicity in sugarcane plantlets grown on acidic soils of southern China. *PLoS One* 11:e0148956. 10.1371/journal.pone.0148956 27023702 PMC4811547

[B86] Husted S. Laursen K. H. Hebbern C. A. Schmidt S. B. Pedas P. Haldrup A. (2009). Manganese deficiency leads to genotype-specific changes in fluorescence induction kinetics and state transitions. *Plant Physiol.* 150 825–833. 10.1104/pp.108.134601 19369593 PMC2689976

[B87] Ihnatowicz A. Siwinska J. Meharg A. A. Carey M. Koornneef M. Reymond M. (2014). Conserved histidine of metal transporter AtNRAMP1 is crucial for optimal plant growth under manganese deficiency at chilling temperatures. *New Phytol.* 202 1173–1183. 10.1111/nph.12737 24571269

[B88] Ishimaru Y. Masuda H. Bashir K. Inoue H. Tsukamoto T. Takahashi M. (2010). Rice metal-nicotianamine transporter, OsYSL2, is required for the long-distance transport of iron and manganese. *Plant J.* 62 379–390. 10.1111/j.1365-313X.2010.04158.x 20128878

[B89] Ishimaru Y. Takahashi R. Bashir K. Shimo H. Senoura T. Sugimoto K. (2012). Characterizing the role of rice NRAMP5 in manganese, iron and cadmium transport. *Sci. Rep.* 2:286. 10.1038/srep00286 22368778 PMC3285952

[B90] Jauregui M. A. Reisenauer H. M. (1982). Dissolution of oxides of manganese and iron by root exudate components. *Soil Sci. Soc. Am. J.* 46 314–317. 10.2136/sssaj1982.03615995004600020020x

[B91] Johnson N. A. Liu F. Weeks P. D. Hentzen A. E. Kruse H. P. Parker J. J. (2009). A tomato ER-type Ca^2 +^-ATPase, LCA1, has a low thapsigargin-sensitivity and can transport manganese. *Arch. Biochem. Biophys.* 481 157–168. 10.1016/j.abb.2008.11.010 19056336

[B92] Kamer K. J. Sancak Y. Fomina Y. Meisel J. D. Chaudhuri D. Grabarek Z. (2018). MICU1 imparts the mitochondrial uniporter with the ability to discriminate between Ca^2 +^ and Mn^2 +^. *Proc. Natl. Acad. Sci. U.S.A.* 115 E7960–E7969. 10.1073/pnas.1807811115 30082385 PMC6112746

[B93] Kamiya T. Akahori T. Ashikari M. Maeshima M. (2006). Expression of the vacuolar Ca^2 +^/H^+^ exchanger, OsCAX1a, in rice: cell and age specificity of expression, and enhancement by Ca^2 +^. *Plant Cell Physiol.* 47 96–106. 10.1093/pcp/pci227 16275657

[B94] Kamiya T. Akahori T. Maeshima M. (2005). Expression profile of the genes for rice cation/H^+^ exchanger family and functional analysis in yeast. *Plant Cell Physiol.* 46 1735–1740. 10.1093/pcp/pci173 16043430

[B95] Kamrul Huda K. M. Yadav S. Akhter Banu M. S. Trivedi D. K. Tuteja N. (2013). Genome-wide analysis of plant-type II Ca^2 +^ ATPases gene family from rice and Arabidopsis: potential role in abiotic stresses. *Plant Physiol. Biochem.* 65 32–47. 10.1016/j.plaphy.2013.01.002 23416494

[B96] Khabaz-Saberi H. Setter T. L. Waters I. (2006). Waterlogging induces high to toxic concentrations of iron, aluminum, and manganese in wheat varieties on acidic soil. *J. Plant Nutr.* 29 899–911. 10.1080/01904160600649161

[B97] Kim K. N. Lee J. S. Han H. Choi S. A. Go S. J. Yoon I. S. (2003). Isolation and characterization of a novel rice Ca^2 +^-regulated protein kinase gene involved in responses to diverse signals including cold, light, cytokinins, sugars and salts. *Plant Mol. Biol.* 52 1191–1202. 10.1023/b:plan.0000004330.62660.a2 14682618

[B98] Kim S. A. Punshon T. Lanzirotti A. Li L. Alonso J. M. Ecker J. R. (2006). Localization of iron in *Arabidopsis* seed requires the vacuolar membrane transporter VIT1. *Science* 314 1295–1298. 10.1126/science.1132563 17082420

[B99] Kirichok Y. Krapivinsky G. Clapham D. E. (2004). The mitochondrial calcium uniporter is a highly selective ion channel. *Nature* 427 360–364. 10.1038/nature02246 14737170

[B100] Koike S. Inoue H. Mizuno D. Takahashi M. Nakanishi H. Mori S. (2004). OsYSL2 is a rice metal-nicotianamine transporter that is regulated by iron and expressed in the phloem. *Plant J.* 39 415–424. 10.1111/j.1365-313x.2004.02146.x 15255870

[B101] Köllner T. G. Held M. Lenk C. Hiltpold I. Turlings T. C. J. Gershenzon J. (2008). A maize (E)-β-caryophyllene synthase implicated in indirect defense responses against herbivores is not expressed in most American maize varieties. *Plant Cell* 20 482–494. 10.1105/tpc.107.051672 18296628 PMC2276456

[B102] Koren’kov V. Park S. Cheng N. H. Sreevidya C. Lachmansingh J. Morris J. (2007). Enhanced Cd^2 +^-selective root-tonoplast-transport in tobaccos expressing Arabidopsis cation exchangers. *Planta* 225 403–411. 10.1007/s00425-006-0352-7 16845524

[B103] Korshunova Y. O. Eide D. Clark W. G. Guerinot M. L. Pakrasi H. B. (1999). The IRT1 protein from *Arabidopsis thaliana* is a metal transporter with a broad substrate range. *Plant Mol. Biol.* 40 37–44. 10394943 10.1023/a:1026438615520

[B104] Lambers H. Hayes P. E. Laliberté E. Oliveira R. S. Turner B. L. (2015). Leaf manganese accumulation and phosphorus-acquisition efficiency. *Trends Plant Sci.* 20 83–90. 10.1016/j.tplants.2014.10.007 25466977

[B105] Lane B. G. (2002). Oxalate, germins, and higher-plant pathogens. *IUBMB Life* 53 67–75. 10.1080/15216540211474 12049198

[B106] Lanquar V. Lelièvre F. Barbier-Brygoo H. Thomine S. (2004). Regulation and function of AtNRAMP4 metal transporter protein. *Soil Sci. Plant Nutr.* 50 1141–1150. 10.1080/00380768.2004.10408587

[B107] Lanquar V. Lelièvre F. Bolte S. Hamès C. Alcon C. Neumann D. (2005). Mobilization of vacuolar iron by AtNRAMP3 and AtNRAMP4 is essential for seed germination on low iron. *EMBO J.* 24 4041–4051. 10.1038/sj.emboj.7600864 16270029 PMC1356305

[B108] Lanquar V. Schnell Ramos M. Lelièvre F. Barbier-Brygoo H. Krieger-Liszkay A. Krämer U. (2010). Export of vacuolar manganese by AtNRAMP3 and AtNRAMP4 is required for optimal photosynthesis and growth under manganese deficiency. *Plant Physiol.* 152 1986–1999. 10.1104/pp.109.150946 20181755 PMC2850043

[B109] LeClere S. Tellez R. Rampey R. A. Matsuda S. P. T. Bartel B. (2002). Characterization of a family of IAA-amino acid conjugate hydrolases from *Arabidopsis*. *J. Biol. Chem.* 277 20446–20452. 10.1074/jbc.m111955200 11923288

[B110] Lešková A. Giehl R. F. H. Hartmann A. Fargašová A. von Wirén N. (2017). Heavy metals induce iron deficiency responses at different hierarchic and regulatory levels. *Plant Physiol.* 174 1648–1668. 10.1104/pp.16.01916 28500270 PMC5490887

[B111] Li C. Wang P. Menzies N. W. Lombi E. Kopittke P. M. (2017). Effects of changes in leaf properties mediated by methyl jasmonate (MeJA) on foliar absorption of Zn, Mn and Fe. *Ann. Bot.* 120 405–415. 10.1093/aob/mcx063 28641371 PMC5591425

[B112] Li J. Y. Liu J. Dong D. Jia X. Mccouch S. R. Kochian L. V. (2014). Natural variation underlies alterations in Nramp aluminum transporter (NRAT1) expression and function that play a key role in rice aluminum tolerance. *Proc. Natl. Acad. Sci. U.S.A.* 111 6503–6508. 10.1073/pnas.1318975111 24728832 PMC4035919

[B113] Li L. Xu X. Chen C. Shen Z. (2016). Genome-wide characterization and expression analysis of the Germin-like protein family in rice and Arabidopsis. *Int. J. Mol. Sci.* 17:1622. 10.3390/ijms17101622 27669230 PMC5085655

[B114] Li L. Yang X. (2018). The essential element manganese, oxidative stress, and metabolic diseases: links and interactions. *Oxid. Med. Cell. Longev.* 2018:7580707. 10.1155/2018/7580707 29849912 PMC5907490

[B115] Li Q. Chen L. S. Jiang H. X. Tang N. Yang L. T. Lin Z. H. (2010). Effects of manganese-excess on CO_2_ assimilation, ribulose-1,5-bisphosphate carboxylase/oxygenase, carbohydrates and photosynthetic electron transport of leaves, and antioxidant systems of leaves and roots in *Citrus grandis* seedlings. *BMC Plant Biol.* 10:42. 10.1186/1471-2229-10-42 20205939 PMC2848762

[B116] Li X. Chanroj S. Wu Z. Romanowsky S. M. Harper J. F. Sze H. (2008). A distinct endosomal Ca^2 +^/Mn^2 +^ pump affects root growth through the secretory process. *Plant Physiol.* 147 1675–1689. 10.1104/pp.108.119909 18567829 PMC2492598

[B117] Liang F. Cunningham K. W. Harper J. F. Sze H. (1997). ECA1 complements yeast mutants defective in Ca^2 +^ pumps and encodes an endoplasmic reticulum-type Ca^2 +^-ATPase in *Arabidopsis thaliana*. *Proc. Natl. Acad. Sci. U.S.A.* 94 8579–8584. 10.1073/pnas.94.16.8579 9238019 PMC23025

[B118] Lidon F. C. Barreiro M. G. Ramalho J. C. (2004). Manganese accumulation in rice: implications for photosynthetic functioning. *J. Plant Physiol.* 161 1235–1244. 10.1016/j.jplph.2004.02.003 15602815

[B119] Liu J. Gao Y. Tang Y. Wang D. Chen X. Yao Y. (2019). Genome-wide identification, comprehensive gene feature, evolution, and expression analysis of plant metal tolerance proteins in tobacco under heavy metal toxicity. *Front. Genet.* 10:345. 10.3389/fgene.2019.00345 31105736 PMC6491887

[B120] Long L. Persson D. P. Duan F. Jørgensen K. Yuan L. Schjoerring J. K. (2018). The iron-regulated transporter 1 plays an essential role in uptake, translocation and grain-loading of manganese, but not iron, in barley. *New Phytol.* 217 1640–1653. 10.1111/nph.14930 29206303

[B121] Longnecker N. E. Graham R. D. Card G. (1991). Effects of manganese deficiency on the pattern of tillering and development of barley (*Hordeum vulgare* c.v. galleon). *Field Crops Res.* 28 85–102. 10.1016/0378-4290(91)90076-8

[B122] López-Millán A. F. Ellis D. R. Grusak M. A. (2004). Identification and characterization of several new members of the ZIP family of metal ion transporters in *Medicago truncatula*. *Plant Mol. Biol.* 54 583–596. 10.1023/b:plan.0000038271.96019.aa 15316291

[B123] Lovely D. R. (1995). Microbial reduction of iron, manganese, and other metals. *Adv. Agron.* 54 175–231. 10.1016/s0065-2113(08)60900-1

[B124] Ma G. Li J. Li J. Li Y. Gu D. Chen C. (2018). OsMTP11, a trans-Golgi network localized transporter, is involved in manganese tolerance in rice. *Plant Sci.* 274 59–69. 10.1016/j.plantsci.2018.05.011 30080641

[B125] Martinoia E. Meyer S. De Angeli A. Nagy R. (2012). Vacuolar transporters in their physiological context. *Annu. Rev. Plant Biol.* 63 183–213. 10.1146/annurev-arplant-042811-105608 22404463

[B126] Martins V. Carneiro F. Conde C. Sottomayor M. Gerós H. (2017). The grapevine VvCAX3 is a cation/H^+^ exchanger involved in vacuolar Ca^2 +^ homeostasis. *Planta* 246 1083–1096. 10.1007/s00425-017-2754-0 28801786

[B127] Matsushima R. Hayashi Y. Yamada K. Shimada T. Nishimura M. Hara-Nishimura I. (2003). The ER body, a novel endoplasmic reticulum-derived structure in *Arabidopsis*. *Plant Cell Physiol.* 44 661–666. 10.1093/pcp/pcg089 12881493

[B128] McNaughton R. L. Reddi A. R. Clement M. H. S. Sharma A. Barnese K. Rosenfeld L. (2010). Probing *in vivo* Mn^2 +^ speciation and oxidative stress resistance in yeast cells with electron-nuclear double resonance spectroscopy. *Proc. Natl. Acad. Sci U.S.A.* 107 15335–15339. 10.1073/pnas.1009648107 20702768 PMC2932569

[B129] McNear D. H. Küpper J. V. (2014). Mechanisms of trichome-specific Mn accumulation and toxicity in the Ni hyperaccumulator *Alyssum murale*. *Plant Soil* 377 407–422. 10.1007/s11104-013-2003-7

[B130] Mei H. Cheng N. H. Zhao J. Park S. Escareno R. A. Pittman J. K. (2009). Root development under metal stress in *Arabidopsis thaliana* requires the H^+^/cation antiporter CAX4. *New Phytol.* 183 95–105. 10.1111/j.1469-8137.2009.02831.x 19368667

[B131] Meinhard M. Rodriguez P. L. Grill E. (2002). The sensitivity of ABI2 to hydrogen peroxide links the abscisic acid-response regulator to redox signalling. *Planta* 214 775–782. 10.1007/s00425-001-0675-3 11882947

[B132] Membré N. Bernier F. Staiger D. Berna A. (2000). *Arabidopsis thaliana* germin-like proteins: common and specific features point to a variety of functions. *Planta* 211 345–354. 10.1007/s004250000277 10987552

[B133] Meng J. G. Zhang X. D. Tan S. K. Zhao K. X. Yang Z. M. (2017). Genome-wide identification of Cd-responsive NRAMP transporter genes and analyzing expression of NRAMP1 mediated by miR167 in *Brassica napus*. *Biometals* 30 917–931. 10.1007/s10534-017-0057-3 28993932

[B134] Migocka M. Papierniak A. Kosieradzka A. Posyniak E. Maciaszczyk-Dziubinska E. Biskup R. (2015). Cucumber metal tolerance protein CsMTP9 is a plasma membrane H^+^-coupled antiporter involved in the Mn^2 +^ and Cd^2 +^ efflux from root cells. *Plant J.* 84 1045–1058. 10.1111/tpj.13056 26485215

[B135] Millaleo R. Reyes-Díaz M. Alberdi M. Ivanov A. G. Krol M. Hüner N. P. A. (2013). Excess manganese differentially inhibits photosystem I versus II in *Arabidopsis thaliana*. *J. Exp. Bot.* 64 343–354. 10.1093/jxb/ers339 23183256 PMC3528040

[B136] Millaleo R. Reyes-Diaz M. Ivanov A. G. Mora M. L. Alberdi M. (2010). Manganese as essential and toxic element for plants: transport, accumulation and resistance mechanisms. *J. Soil Sci. Plant Nutr.* 10 470–481. 10.4067/s0718-95162010000200008

[B137] Mills R. F. Doherty M. L. Lopez-Marques R. L. Weimar T. Dupree P. Palmgren M. G. (2007). ECA3, a Golgi-localized P_2A_-type ATPase, plays a crucial role in manganese nutrition in Arabidopsis. *Plant Physiol.* 146 116–128. 10.1104/pp.107.110817 18024560 PMC2230566

[B138] Milner M. J. Seamon J. Craft E. Kochian L. V. (2013). Transport properties of members of the ZIP family in plants and their role in Zn and Mn homeostasis. *J. Exp. Bot.* 64 369–381. 10.1093/jxb/ers315 23264639 PMC3528025

[B139] Montanini B. Blaudez D. Jeandroz S. Sanders D. Chalot M. (2007). Phylogenetic and functional analysis of the Cation Diffusion Facilitator (CDF) family: improved signature and prediction of substrate specificity. *BMC Genomics* 8:107. 10.1186/1471-2164-8-107 17448255 PMC1868760

[B140] Morgan J. J. (2005). Kinetics of reaction between O_2_ and Mn(II) species in aqueous solutions. *Geochim. Cosmochim. Acta* 69 35–48. 10.1016/j.gca.2004.06.013

[B141] Morgan P. W. Joham H. E. Amin J. V. (1966). Effect of manganese toxicity on the indoleacetic acid oxidase system of cotton. *Plant Physiol.* 41 718–724. 10.1104/pp.41.4.718 16656311 PMC1086411

[B142] Morris J. Tian H. Park S. Sreevidya C. S. Ward J. M. Hirschi K. D. (2008). AtCCX3 is an Arabidopsis endomembrane H^+^-dependent K^+^ transporter. *Plant Physiol.* 148 1474–1486. 10.1104/pp.108.118810 18775974 PMC2577254

[B143] Nable R. O. Houtz R. L. Cheniae G. M. (1988). Early inhibition of photosynthesis during development of Mn toxicity in tobacco. *Plant Physiol.* 86 1136–1142. 10.1104/pp.86.4.1136 16666045 PMC1054641

[B144] Nevo Y. Nelson N. (2006). The NRAMP family of metal-ion transporters. *Biochim. Biophys. Acta Mol. Cell Res.* 1763 609–620. 10.1016/j.bbamcr.2006.05.007 16908340

[B145] Nian L. S. (1989). Manganese induced iron chlorosis in pineapple and its control by foliar application of iron-citrate. *J. Plant Nutr. Soil Sci.* 152 125–126. 10.1002/jpln.19891520122

[B146] Nowicki M. Müller F. Frentzen M. (2005). Cardiolipin synthase of *Arabidopsis thaliana*. *FEBS Lett.* 579 2161–2165. 10.1016/j.febslet.2005.03.007 15811335

[B147] Nuruzzaman M. Lambers H. Bolland M. D. A. Veneklaas E. J. (2006). Distribution of carboxylates and acid phosphatase and depletion of different phosphorus fractions in the rhizosphere of a cereal and three grain legumes. *Plant Soil* 281 109–120. 10.1007/s11104-005-3936-2

[B148] Ohki K. (1985). Manganese deficiency and toxicity effects on photosynthesis, chlorophyll, and transpiration in Wheat. *Crop Sci.* 25 187–191. 10.2135/cropsci1985.0011183x002500010045x

[B149] Önnerud H. Zhang L. Gellerstedt G. Henriksson G. (2002). Polymerization of monolignols by redox shuttle–mediated enzymatic oxidation. *Plant Cell* 14 1953–1962. 10.1105/tpc.001487 12172033 PMC151476

[B150] Oomen R. J. F. J. Wu J. Lelièvre F. Blanchet S. Richaud P. Barbier-Brygoo H. (2009). Functional characterization of NRAMP3 and NRAMP4 from the metal hyperaccumulator *Thlaspi caerulescens*. *New Phytol.* 181 637–650. 10.1111/j.1469-8137.2008.02694.x 19054339

[B151] Panda S. Mishra A. K. Biswal U. C. (1987). Manganese induced peroxidation of thylakoid lipids and changes in chlorophyll-α fluorescence during aging of cell free chloroplasts in light. *Phytochemistry* 26 3217–3219. 10.1016/s0031-9422(00)82472-3

[B152] Papadakis I. E. Giannakoula A. Therios I. N. Bosabalidis A. M. Moustakas M. Nastou A. (2007). Mn-induced changes in leaf structure and chloroplast ultrastructure of *Citrus volkameriana* (L.) plants. *J. Plant Physiol.* 164 100–103. 10.1016/j.jplph.2006.04.011 16781796

[B153] Pedas P. Hebbern C. A. Schjoerring J. K. Holm P. E. Husted S. (2005). Differential capacity for high-affinity manganese uptake contributes to differences between barley genotypes in tolerance to low manganese availability. *Plant Physiol.* 139 1411–1420. 10.1104/pp.105.067561 16244151 PMC1283776

[B154] Pedas P. Husted S. Skytte K. Schjoerring J. K. (2011). Elevated phosphorus impedes manganese acquisition by barley plants. *Front. Plant Sci.* 2:37. 10.3389/fpls.2011.00037 22639592 PMC3355622

[B155] Pedas P. Stokholm M. S. Hegelund J. N. Ladegård A. H. Schjoerring J. K. Husted S. (2014). Golgi localized barley MTP8 proteins facilitate Mn transport. *PLoS One* 9:e113759. 10.1371/journal.pone.0113759 25486417 PMC4259309

[B156] Pedas P. Ytting C. K. Fuglsang A. T. Jahn T. P. Schjoerring J. K. Husted S. (2008). Manganese efficiency in barley: identification and characterization of the metal ion transporter HvIRT1. *Plant Physiol.* 148 455–466. 10.1104/pp.108.118851 18614714 PMC2528110

[B157] Peiter E. Montanini B. Gobert A. Pedas P. Husted S. Maathuis F. J. M. (2007). A secretory pathway-localized cation diffusion facilitator confers plant manganese tolerance. *Proc. Natl. Acad. Sci. U.S.A.* 104 8532–8537. 10.1073/pnas.0609507104 17494768 PMC1895984

[B158] Peris-Peris C. Serra-Cardona A. Sánchez-Sanuy F. Campo S. Ariño J. San Segundo B. (2017). Two NRAMP6 isoforms function as iron and manganese transporters and contribute to disease resistance in rice. *Mol. Plant Microbe Interact.* 30 385–398. 10.1094/MPMI-01-17-0005-R 28430017

[B159] Pittman J. K. (2005). Managing the manganese: molecular mechanisms of manganese transport and homeostasis. *New Phytol.* 167 733–742. 10.1111/j.1469-8137.2005.01453.x 16101910

[B160] Pittman J. K. Hirschi K. D. (2016). Phylogenetic analysis and protein structure modelling identifies distinct Ca^2 +^/cation antiporters and conservation of gene family structure within Arabidopsis and rice species. *Rice* 9:3 10.1186/s12284-016-0075-8 PMC4735048 26833031

[B161] Pittman J. K. Shigaki T. Marshall J. L. Morris J. L. Cheng N. Hirschi K. D. (2004). Functional and regulatory analysis of the *Arabidopsis thaliana* CAX2 cation transporter. *Plant Mol. Biol.* 56 959–971. 10.1007/s11103-004-6446-3 15821993

[B162] Pottier M. Oomen R. Picco C. Giraudat J. Scholz-Starke J. Richaud P. (2015). Identification of mutations allowing natural resistance associated macrophage proteins (NRAMP) to discriminate against cadmium. *Plant J.* 83 625–637. 10.1111/tpj.12914 26088788

[B163] Reddi A. R. Jensen L. T. Naranuntarat A. Rosenfeld L. Leung E. Shah R. (2009). The overlapping roles of manganese and Cu/Zn SOD in oxidative stress protection. *Free Radic. Biol. Med.* 46 154–162. 10.1016/j.freeradbiomed.2008.09.032 18973803 PMC2707084

[B164] Rengel Z. (2015). Availability of Mn, Zn and Fe in the rhizosphere. *J. Soil Sci. Plant Nutr.* 15 397–409.

[B165] Requena L. Bornemann S. (1999). Barley (*Hordeum vulgare*) oxalate oxidase is a manganese-containing enzyme. *Biochem. J.* 343 185–190. 10.1042/bj3430185 10493928 PMC1220540

[B166] Robert H. S. Offringa R. (2008). Regulation of auxin transport polarity by AGC kinases. *Curr. Opin. Plant Biol.* 11 495–502. 10.1016/j.pbi.2008.06.004 18640868

[B167] Rodríguez-Celma J. Tsai Y.-H. Wen T.-N. Wu Y.-C. Curie C. Schmidt W. (2016). Systems-wide analysis of manganese deficiency-induced changes in gene activity of *Arabidopsis* roots. *Sci. Rep.* 6 35846–35846. 10.1038/srep35846 27804982 PMC5090222

[B168] Rohdich F. Lauw S. Kaiser J. Feicht R. Köhler P. Bacher A. (2006). Isoprenoid biosynthesis in plants - 2C-methyl-D-erythritol-4-phosphate synthase (IspC protein) of *Arabidopsis thaliana*. *FEBS J.* 273 4446–4458. 10.1111/j.1742-4658.2006.05446.x 16972937

[B169] Rohdich F. Wungsintaweekul J. Eisenreich W. Richter G. Schuhr C. A. Hecht S. (2000). Biosynthesis of terpenoids: 4-diphosphocytidyl-2C-methyl-D-erythritol synthase of *Arabidopsis thaliana*. *Proc. Natl. Acad. Sci. U.S.A.* 97 6451–6456. 10.1073/pnas.97.12.6451 10841550 PMC18623

[B170] Sasaki A. Yamaji N. Xia J. Ma J. F. (2011). OsYSL6 is involved in the detoxification of excess manganese in rice. *Plant Physiol.* 157 1832–1840. 10.1104/pp.111.186031 21969384 PMC3327210

[B171] Sasaki A. Yamaji N. Yokosho K. Ma J. F. (2012). Nramp5 is a major transporter responsible for manganese and cadmium uptake in rice. *Plant Cell* 24 2155–2167. 10.1105/tpc.112.096925 22589467 PMC3442593

[B172] Schaaf G. Ludewig U. Erenoglu B. E. Mori S. Kitahara T. von Wirén N. (2004). ZmYS1 functions as a proton-coupled symporter for phytosiderophore- and nicotianamine-chelated metals. *J. Biol. Chem.* 279 9091–9096. 10.1074/jbc.m311799200 14699112

[B173] Schenk G. Ge Y. Carrington L. E. Wynne C. J. Searle I. R. Carroll B. J. (1999). Binuclear metal centers in plant purple acid phosphatases: Fe-Zn in sweet potato and Fe-Zn in soybean. *Arch. Biochem. Biophys.* 370 183–189. 10.1006/abbi.1999.1407 10510276

[B174] Schmidt S. B. Jensen P. E. Husted S. (2016). Manganese deficiency in plants: The impact on photosystem II. *Trends Plant Sci.* 21 622–632. 10.1016/j.tplants.2016.03.001 27150384

[B175] Schneider A. Steinberger I. Herdean A. Gandini C. Eisenhut M. Kurz S. (2016). The evolutionarily conserved protein PHOTOSYNTHESIS AFFECTED MUTANT71 is required for efficient manganese uptake at the thylakoid membrane in Arabidopsis. *Plant Cell* 28 892–910. 10.1105/tpc.15.00812 27020959 PMC4863382

[B176] Schweighofer A. Hirt H. Meskiene I. (2004). Plant PP2C phosphatases: Emerging functions in stress signaling. *Trends Plant Sci.* 9 236–243. 10.1016/j.tplants.2004.03.007 15130549

[B177] Sello S. Moscatiello R. Mehlmer N. Leonardelli M. Carraretto L. Cortese E. (2018). Chloroplast Ca^2 +^ fluxes into and across thylakoids revealed by thylakoid-targeted aequorin probes. *Plant Physiol.* 177 38–51. 10.1104/pp.18.00027 29559589 PMC5933129

[B178] Shao J. F. Yamaji N. Shen R. F. Ma J. F. (2017). The key to Mn homeostasis in plants: regulation of Mn transporters. *Trends Plant Sci.* 22 215–224. 10.1016/j.tplants.2016.12.005 28087151

[B179] Shigaki T. Pittman J. K. Hirschi K. D. (2003). Manganese specificity determinants in the *Arabidopsis* metal/H^+^ antiporter CAX2. *J. Biol. Chem.* 278 6610–6617. 10.1074/jbc.M209952200 12496310

[B180] Socha A. L. Guerinot M. L. (2014). Mn-euvering manganese: the role of transporter gene family members in manganese uptake and mobilization in plants. *Front. Plant Sci.* 5:106. 10.3389/fpls.2014.00106 24744764 PMC3978347

[B181] Sparrow L. A. Uren N. C. (2014). Manganese oxidation and reduction in soils: effects of temperature, water potential, pH and their interactions. *Soil Res.* 52 483–494. 10.1071/SR13159

[B182] St.Clair S. B. Lynch J. P. (2005). Element accumulation patterns of deciduous and evergreen tree seedlings on acid soils: implications for sensitivity to manganese toxicity. *Tree Physiol.* 25 85–92. 10.1093/treephys/25.1.85 15519989

[B183] Stael S. Wurzinger B. Mair A. Mehlmer N. Vothknecht U. C. Teige M. (2012). Plant organellar calcium signalling: an emerging field. *J. Exp. Bot.* 63 1525–1542. 10.1093/jxb/err394 22200666 PMC3966264

[B184] Stengel A. Gügel I. L. Hilger D. Rengstl B. Jung H. Nickelsen J. (2012). Initial steps of photosystem II de novo assembly and preloading with manganese take place in biogenesis centers in Synechocystis. *Plant Cell* 24 660–675. 10.1105/tpc.111.093914 22319052 PMC3315239

[B185] Stoltz E. Wallenhammar A. C. (2014). Manganese application increases winter hardiness in barley. *Field Crops Res.* 164 148–153. 10.1016/j.fcr.2014.05.008

[B186] Strasser R. Bondili J. S. Vavra U. Schoberer J. Svoboda B. Glossl J. (2007). A unique β1,3-galactosyltransferase is indispensable for the biosynthesis of N-glycans containing Lewis a structures in *Arabidopsis thaliana*. *Plant Cell* 19 2278–2292. 10.1105/tpc.107.052985 17630273 PMC1955701

[B187] Stumm W. Morgan J. J. (1996). *Aquatic Chemistry: Chemical Equilibria and Rates in Natural Waters.* New York, NY: Wiley.

[B188] Subrahmanyam D. Rathore V. S. (2001). Influence of manganese toxicity on photosynthesis in ricebean (*Vigna umbellata*) seedlings. *Photosynthetica* 38 449–453.

[B189] Svedruzic D. Jonsson S. Toyota C. G. Reinhardt L. A. Ricagno S. Lindqvist Y. (2005). The enzymes of oxalate metabolism: unexpected structures and mechanisms. *Arch. Biochem. Biophys.* 433 176–192. 10.1016/j.abb.2004.08.032 15581576

[B190] Szurmak B. Wysłouch-Cieszyńska A. Wszelaka-Rylik M. Bal W. Dobrzańska M. (2008). A diadenosine 5′,5″-P1P4 tetraphosphate (Ap_4_A) hydrolase from *Arabidopsis thaliana* that is activated preferentially by Mn^2 +^ ions. *Acta Biochim. Polon.* 55 151–160. 10.18388/abp.2008_3173 18345354

[B191] Takahashi S. Sakamoto A. N. Tanaka A. Shimizu K. (2007). AtREV1, a Y-family DNA polymerase in Arabidopsis, has deoxynucleotidyl transferase activity *in vitro*. *Plant Physiol.* 145 1052–1060. 10.1104/pp.107.101980 17827267 PMC2048784

[B192] Takemoto Y. Tsunemitsu Y. Fujii-Kashino M. Mitani-Ueno N. Yamaji N. Ma J. F. (2017). The tonoplast-localized transporter MTP8.2 contributes to manganese detoxification in the shoots and roots of *Oryza sativa* L. *Plant Cell Physiol.* 58 1573–1582. 10.1093/pcp/pcx082 28633293

[B193] Teardo E. Carraretto L. Moscatiello R. Cortese E. Vicario M. Festa M. (2019). A chloroplast-localized mitochondrial calcium uniporter transduces osmotic stress in *Arabidopsis*. *Nat. Plants* 5 581–588. 10.1038/s41477-019-0434-8 31182842

[B194] Thomine S. Lelièvre F. Debarbieux E. Schroeder J. I. Barbier-Brygoo H. (2003). AtNRAMP3, a multispecific vacuolar metal transporter involved in plant responses to iron deficiency. *Plant J.* 34 685–695. 10.1046/j.1365-313X.2003.01760.x 12787249

[B195] Thomine S. Wang R. Ward J. M. Crawford N. M. Schroeder J. I. (2000). Cadmium and iron transport by members of a plant metal transporter family in *Arabidopsis* with homology to *Nramp* genes. *Proc. Natl. Acad. Sci. U.S.A.* 97 4991–4996. 10.1073/pnas.97.9.4991 10781110 PMC18345

[B196] Tiwari M. Sharma D. Dwivedi S. Singh M. Tripathi R. D. Trivedi P. K. (2014). Expression in *Arabidopsis* and cellular localization reveal involvement of rice NRAMP, OsNRAMP1, in arsenic transport and tolerance. *Plant Cell Environ.* 37 140–152. 10.1111/pce.12138 23700971

[B197] Tsukamoto T. Nakanishi H. Kiyomiya S. Watanabe S. Matsuhashi S. Nishizawa N. K. (2006). ^52^Mn translocation in barley monitored using a positron-emitting tracer imaging system. *Soil Sci. Plant Nutr.* 52 717–725. 10.1111/j.1747-0765.2006.00096.x

[B198] Tsunemitsu Y. Genga M. Okada T. Yamaji N. Ma J. F. Miyazaki A. (2018). A member of cation diffusion facilitator family, MTP11, is required for manganese tolerance and high fertility in rice. *Planta* 248 231–241. 10.1007/s00425-018-2890-1 29700611

[B199] Turekian K. K. Wedepohl K. H. (1961). Distribution of the elements in some major units of the earth’s crust. *GSA Bull.* 72 175–192.

[B200] Ueno D. Sasaki A. Yamaji N. Miyaji T. Fujii Y. Takemoto Y. (2015). A polarly localized transporter for efficient manganese uptake in rice. *Nat. Plants* 1:15170. 10.1038/nplants.2015.170 27251715

[B201] Ullah I. Wang Y. Eide D. J. Dunwell J. M. (2018). Evolution, and functional analysis of natural resistance-associated macrophage proteins (NRAMPs) from *Theobroma cacao* and their role in cadmium accumulation. *Sci. Rep.* 8:14412. 10.1038/s41598-018-32819-y 30258092 PMC6158261

[B202] Venkidasamy B. Selvaraj D. Ramalingam S. (2019). Genome-wide analysis of purple acid phosphatase (PAP) family proteins in *Jatropha curcas* L. *Int. J. Biol. Macromol.* 123 648–656. 10.1016/j.ijbiomac.2018.11.027 30414420

[B203] Vert G. Grotz N. Dédaldéchamp F. Gaymard F. Guerinot M. Lou B. J. (2002). IRT1, an Arabidopsis transporter essential for iron uptake from the soil and for plant growth. *Plant Cell* 14 1223–1233. 10.1105/tpc.001388 12084823 PMC150776

[B204] Wagner S. Behera S. De Bortoli S. Logan D. C. Fuchs P. Carraretto L. (2015). The EF-hand Ca^2 +^ binding protein MICU choreographs mitochondrial Ca^2 +^ dynamics in Arabidopsis. *Plant Cell* 27 3190–3212. 10.1105/tpc.15.00509 26530087 PMC4682298

[B205] Wang C. Guan Y. Lv M. Zhang R. Guo Z. Wei X. (2018). Manganese increases the sensitivity of the cGAS-STING pathway for double-stranded DNA and is required for the host defense against DNA viruses. *Immunity* 48 675.e7–687.e7. 10.1016/j.immuni.2018.03.017 29653696

[B206] Wang C. Xu W. Jin H. Zhang T. Lai J. Zhou X. (2016). A putative chloroplast-localized Ca^2 +^/H^+^ antiporter CCHA1 is involved in calcium and pH homeostasis and required for PSII function in *Arabidopsis*. *Mol. Plant* 9 1183–1196. 10.1016/j.molp.2016.05.015 27302341

[B207] Wang N. Qiu W. Dai J. Guo X. Lu Q. Wang T. (2019). AhNRAMP1 enhances manganese and zinc uptake in plants. *Front. Plant Sci.* 10:415. 10.3389/fpls.2019.00415 31134101 PMC6514220

[B208] Wang X. Zhong F. Woo C. H. Miao Y. Grusak M. A. Zhang X. (2017). A rapid and efficient method to study the function of crop plant transporters in *Arabidopsis*. *Protoplasma* 254 737–747. 10.1007/s00709-016-0987-6 27240439

[B209] Waters B. M. Chu H. H. Didonato R. J. Roberts L. A. Eisley R. B. Lahner B. (2006). Mutations in Arabidopsis *Yellow Stripe-Like1* and *Yellow Stripe-Like3* reveal their roles in metal ion homeostasis and loading of metal ions in seeds. *Plant Physiol.* 141 1446–1458. 10.1104/pp.106.082586 16815956 PMC1533956

[B210] Werner A. K. Sparkes I. A. Romeis T. Witte C. P. (2008). Identification, biochemical characterization, and subcellular localization of allantoate amidohydrolases from Arabidopsis and soybean. *Plant Physiol.* 146 418–430. 10.1104/pp.107.110809 18065556 PMC2245841

[B211] White P. J. Bowen H. C. Demidchik V. Nichols C. Davies J. M. (2002). Genes for calcium-permeable channels in the plasma membrane of plant root cells. *Biochim. Biophys. Acta Biomembr.* 1564 299–309. 10.1016/S0005-2736(02)00509-6 12175911

[B212] White P. J. Greenwood D. J. (2013). “Properties and management of cationic elements for crop growth,” in *Soil Conditions and Plant Growth*, eds Gregory P. J. Nortcliff S. (Oxford: Blackwell Publishing), 160–194 10.1002/9781118337295.ch6

[B213] Wilkinson R. E. Ohki K. (1988). Influence of manganese deficiency and toxicity on isoprenoid syntheses. *Plant Physiol.* 87 841–846. 10.1104/pp.87.4.841 16666235 PMC1054856

[B214] Wu D. Yamaji N. Yamane M. Kashino-Fujii M. Sato K. Ma J. F. (2016). The HvNramp5 transporter mediates uptake of cadmium and manganese, but not iron. *Plant Physiol.* 172 1899–1910. 10.1104/pp.16.01189 27621428 PMC5100758

[B215] Wu Z. Liang F. Hong B. Young J. C. Sussman M. R. Harper J. F. (2002). An endoplasmic reticulum-bound Ca^2 +^/Mn^2 +^ pump, ECA1, supports plant growth and confers tolerance to Mn^2 +^ stress. *Plant Physiol.* 130 128–137. 10.1104/pp.004440 12226493 PMC166546

[B216] Wymer C. L. Bibikova T. N. Gilroy S. (1997). Cytoplasmic free calcium distributions during the development of root hairs of *Arabidopsis thaliana*. *Plant J.* 12 427–439. 10.1046/j.1365-313X.1997.12020427.x 9301093

[B217] Xia J. Yamaji N. Kasai T. Ma J. F. (2010). Plasma membrane-localized transporter for aluminum in rice. *Proc. Natl. Acad. Sci. U.S.A.* 107 18381–18385. 10.1073/pnas.1004949107 20937890 PMC2972927

[B218] Xiao H. Yin L. Xu X. Li T. Han Z. (2008). The iron-regulated transporter, MbNRAMP1, isolated from *Malus baccata* is involved in Fe, Mn and Cd trafficking. *Ann. Bot.* 102 881–889. 10.1093/aob/mcn178 18819951 PMC2712396

[B219] Yamada K. Hara-Nishimura I. Nishimura M. (2011). Unique defense strategy by the endoplasmic reticulum body in plants. *Plant Cell Physiol.* 52 2039–2049. 10.1093/pcp/pcr156 22102697

[B220] Yamada K. Nagano A. J. Nishina M. Hara-Nishimura I. Nishimura M. (2013). Identification of two novel endoplasmic reticulum body-specific integral membrane proteins. *Plant Physiol.* 161 108–120. 10.1104/pp.112.207654 23166355 PMC3532245

[B221] Yamada N. Theerawitaya C. Cha-Um S. Kirdmanee C. Takabe T. (2014). Expression and functional analysis of putative vacuolar Ca^2 +^-transporters (CAXs and ACAs) in roots of salt tolerant and sensitive rice cultivars. *Protoplasma* 251 1067–1075. 10.1007/s00709-014-0615-2 24482191

[B222] Yamaji N. Sasaki A. Xia J. X. Yokosho K. Ma J. F. (2013). A node-based switch for preferential distribution of manganese in rice. *Nat. Commun.* 4:2442. 10.1038/ncomms3442 24048172

[B223] Yang M. Zhang W. Dong H. Zhang Y. Lv K. Wang D. (2013). OsNRAMP3 is a vascular bundles-specific manganese transporter that is responsible for manganese distribution in rice. *PLoS One* 8:e83990. 10.1371/journal.pone.0083990 24391861 PMC3877151

[B224] Yang M. Zhang Y. Zhang L. Hu J. Zhang X. Lu K. (2014). OsNRAMP5 contributes to manganese translocation and distribution in rice shoots. *J. Exp. Bot*. 65 4849–4861. 10.1093/jxb/eru259 24963001 PMC4144776

[B225] Yang T. J. W. Perry P. J. Ciani S. Pandian S. Schmidt W. (2008). Manganese deficiency alters the patterning and development of root hairs in *Arabidopsis*. *J. Exp. Bot.* 59 3453–3464. 10.1093/jxb/ern195 18772308 PMC2529234

[B226] Zhang B. Zhang C. Liu C. Jing Y. Wang Y. Jin L. (2018). Inner envelope CHLOROPLAST MANGANESE TRANSPORTER 1 supports manganese homeostasis and phototrophic growth in *Arabidopsis*. *Mol. Plant* 11 943–954. 10.1016/j.molp.2018.04.007 29734003

[B227] Zhang M. Liu B. (2017). Identification of a rice metal tolerance protein OsMTP11 as a manganese transporter. *PLoS One* 12:e0174987. 10.1371/journal.pone.0174987 28394944 PMC5386239

[B228] Zhang Y. Xu Y. H. Yi H. Y. Gong J. M. (2012). Vacuolar membrane transporters OsVIT1 and OsVIT2 modulate iron translocation between flag leaves and seeds in rice. *Plant J.* 72 400–410. 10.1111/j.1365-313X.2012.05088.x 22731699

[B229] Zhang Z. Yin H. Tan W. Koopal L. K. Zheng L. Feng X. (2014). Zn sorption to biogenic bixbyite-like Mn_2_O_3_ produced by *Bacillus* CUA isolated from soil: XAFS study with constraints on sorption mechanism. *Chem. Geol.* 389 82–90. 10.1016/j.chemgeo.2014.09.017 25987886

[B230] Zhao J. Wang W. Zhou H. Wang R. Zhang P. Wang H. (2017). Manganese toxicity inhibited root growth by disrupting auxin biosynthesis and transport in *Arabidopsis*. *Front. Plant Sci.* 8:272. 10.3389/fpls.2017.00272 28316607 PMC5334637

